# Drug ranking using machine learning systematically predicts the efficacy of anti-cancer drugs

**DOI:** 10.1038/s41467-021-22170-8

**Published:** 2021-03-25

**Authors:** Henry Gerdes, Pedro Casado, Arran Dokal, Maruan Hijazi, Nosheen Akhtar, Ruth Osuntola, Vinothini Rajeeve, Jude Fitzgibbon, Jon Travers, David Britton, Shirin Khorsandi, Pedro R. Cutillas

**Affiliations:** 1 Cell Signalling & Proteomics Group, Centre for Genomics & Computational Biology, Barts Cancer Institute, Queen Mary University of London, Charterhouse Square, London, UK; 2 Department of Biological Sciences, National University of Medical Sciences, Rawalpindi, Pakistan; 3 Mass spectrometry Laboratory, Barts Cancer Institute, Queen Mary University of London, Charterhouse Square, London, UK; 4 Personalised Medicine Group, Centre for Genomics & Computational Biology, Barts Cancer Institute, Queen Mary University of London, Charterhouse Square, London, UK; 5 Astra Zeneca Ltd, 1 Francis Crick Avenue, Cambridge Biomedical Campus, Cambridge, UK; 6 Kings College London, London, UK; 7 The Alan Turing Institute, The British Library, 2QR, London, UK; 8 Present Address: Kinomica Ltd, Alderley Park, Alderley Edge, Macclesfield, UK

**Keywords:** Cancer, Machine learning, Predictive medicine

## Abstract

Artificial intelligence and machine learning (ML) promise to transform cancer therapies by accurately predicting the most appropriate therapies to treat individual patients. Here, we present an approach, named Drug Ranking Using ML (DRUML), which uses omics data to produce ordered lists of >400 drugs based on their anti-proliferative efficacy in cancer cells. To reduce noise and increase predictive robustness, instead of individual features, DRUML uses internally normalized distance metrics of drug response as features for ML model generation. DRUML is trained using in-house proteomics and phosphoproteomics data derived from 48 cell lines, and it is verified with data comprised of 53 cellular models from 12 independent laboratories. We show that DRUML predicts drug responses in independent verification datasets with low error (mean squared error < 0.1 and mean Spearman’s rank 0.7). In addition, we demonstrate that DRUML predictions of cytarabine sensitivity in clinical leukemia samples are prognostic of patient survival (Log rank *p* < 0.005). Our results indicate that DRUML accurately ranks anti-cancer drugs by their efficacy across a wide range of pathologies.

## Abstract (web summary)

Artificial intelligence and machine learning promise to transform cancer therapies by accurately predicting the most appropriate drugs to treat individual patients. Here, the authors present an approach which uses omics data to produce ordered lists of drugs based on their effectiveness in decreasing cancer cell proliferation.

## Introduction

Cancers derived from the same tissue of origin and pathological classification exhibit high degrees of genetic and phenotypic variability within individuals^1–3^. In practice, this heterogeneity translates to patients having differential responses to therapy. To address this issue, the field of personalized medicine aims to identify measurable biomarkers which correlate with the efficacy of therapeutic interventions in individuals, allowing clinicians to predict patient responses to these therapies^4^.

Protein biomarkers have been used to direct several targeted cancer therapies for decades. Prime examples include HER2 and estrogen receptor, whose expression predict the responses of breast cancer patients to trastuzumab and tamoxifen, respectively^5^. More recently, the development of robust and affordable next-generation sequencing methods is allowing the identification of genetic markers that predict responses to several targeted drugs. Consequently, the majority of modern precision medicine approaches utilize DNA sequencing methods and other genetic analyses such as the detection of chromosomal rearrangements^6,7^. However, despite success in some therapeutic contexts^8,9^, the identification of response remains challenging for many drugs. This is due to the complex biological landscape of cancer, where multiple biochemical pathways compensate each other and contribute to oncogenic phenotypes^10,11^. Thus, mutations and other genetic abnormalities are often inaccurate at stratification. For example, midostaurin—a small molecule inhibitor of the receptor Fms Related Receptor Tyrosine Kinase 3 (FLT3) that also targets other kinases—was approved in 2017 to treat acute myeloid leukemia (AML) patients positive for FLT3 mutations, even though >40% of FLT3 mutant-positive AML patients fail to respond to midostaurin^12^ and >30% of FLT3 mutant negative could potentially benefit from treatment^13^. Similarly, ~65% of eligible breast cancer patients (namely phosphatidylinositol 3-kinase alpha isoform (PIK3CA) mutant and hormone receptor positive) failed to respond to the PI3K inhibitor alpelisib (BYL-719) in a recent clinical trial^14^. A feature of current companion diagnostics is that these consider biomarkers for a given drug in isolation without taking into account the presence of response markers for other drugs that could also be prescribed for a given patient. Consequently, although patient selection using currently used biomarkers can increase the overall efficacy of a given therapy, their ability to identify optimal treatments is often imprecise for a particular cancer patient.

The application of machine learning (ML) to biomedicine promises to revolutionize how cancers are diagnosed and treated in the future^15,16^. Projects such as the Cancer Target Discovery and Development and Genomics of Drug Sensitivity in Cancer have evaluated ML as a means to predict drug responses by associating genomic features, gene expression patterns and copy number alterations to drug sensitivity^17–21^. However, this approach has not been systematically applied using large scale proteomics and phosphoproteomics data, even though anecdotal evidence suggests that proteomic-derived features may be able to predict drug responses more accurately that genomic alternatives^22–26^. A limitation has been the low sample throughput of proteomics and phosphoproteomics by liquid chromatography coupled to tandem mass spectrometry (LC-MS/MS) compared to other omics techniques. Most proteomics methods also involve comparing proteins after chemical or metabolic labeling, thus restricting the number of samples that can be directly compared and used as the input for ML model generation^27^. Moreover, since labeling methods measure protein or phosphorylation sites as ratios, rather than providing absolute values of abundance, models of drug responses constructed with labelled proteomics data may be difficult to validate, and subsequently implement, in verification datasets and in the clinic. Improvements in LC-MS/MS throughput and label-free analysis^28–31^ in tandem, together with the recent availability of systematic drug response profiles for a large number of cell lines and drugs^17,21,32^, now make feasible the use of proteomics and phosphoproteomics data as the input of predictive models of drug responses. Thus, assessing the performance of ML models constructed using proteomics data as input is timely, and essential to evaluate the accuracy and the potential of proteomics to advance the field of precision medicine.

To this end, here, we developed an approach, named Drug Ranking Using ML (DRUML), for building and integrating ML models. DRUML uses combinations of proteomic and phosphoproteomic features to generate lists of ranked drugs based on their efficacy in decreasing cancer cell proliferation. The ability of DRUML to predict drug rankings within a cancer cell population, without the need to compare to reference samples, is crucial for the clinical implementation of ML and fulfills a core aim of precision medicine^6,33^.

## Results

### Overview of approach

**Fig. 1:**
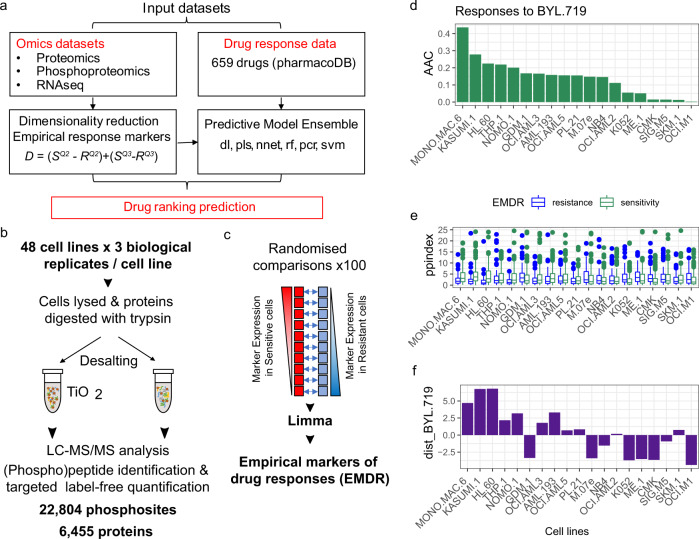
Overview of Drug Ranking Using Machine Learning (DRUML). **a** Drug response (AAC) values were modeled for 659 drugs with different DL/ML methods. Of these, 466 produced empirical markers of drug response and the responses for 411 drugs were reliably modeled by at least one learning algorithm. The input for DL/ML model generation are averaged values of empirical markers of drug responses (EMDRs), which are combined to derive a distance metric *D*. For each drug d and for each biological sample b, D_d,b_ = [(S^Q2^-R^Q2^) + (S^Q3^-R^Q3^)], where S^Q2^ and S^Q3^ are median and third quantile expression values of empirical markers increased in cells sensitive to a given drug, respectively; and R^Q2^ and R^Q3^ are median and third quartile expression values of empirical markers increased in cells resistant to the same drug. **b** LC-MS/MS workflow for the generation of proteomic and phosphoproteomic datasets used to train DRUML. **c** Approach to obtain empirical markers of drug responses (EMDRs). **d** Response values for BYL-719 obtained from PharmacoDB (*n* = 19). To determine empirical markers of drug sensitivity and resistance for BYL-719, cell lines are split into sensitive and resistant groups based on area above curve (AAC) values and Empirical Bayes Statistics of linear models were used to identify response markers by resampling. **e** Boxplot of the distributions of empirical markers of resistance and sensitivity in phosphoproteomics data acquired for the cell lines shown in panel d (measured in triplicate), boxplot with median center, interquartile box boundaries and range upper and lower hinges. **f** BYL-719 *D* values for the named cell lines calculated from the EMDR distributions shown in (**e**). Learning algorithms were random forest (rf), cubist, bayesian estimation of generalized linear models (bglm), partial least squares (pls), principal component regression (pcr), support vector machine (svm), deep learning (dl) and neural network (nnet).

DRUML consists of an ensemble of ML models trained on the responses of cells to >400 drugs, which allows these agents to be ranked based on their predicted efficacy within a sample (Fig. 1a). In principle, any large-scale omics dataset can be used as the input of DRUML. While the use of gene copy number and RNA-seq for the generation of learning models is well documented^18,32,34,35^, the utility and relative performance of large-scale proteomics and phosphoproteomics data is less well explored. Here we used phosphoproteomics and proteomics datasets obtained from 48 AML (*n* = 26), esophagus (*n* = 10) and hepatocellular (*n* = 12) cancer cell lines as the input for DRUML to build models that may be applied to leukemia and solid tumors (Fig. 1b). The predictive accuracy of models, initially trained and validated using these in-house proteomic datasets, were subsequently verified with data obtained from other laboratories (discussed below). Supplementary Fig. 1 shows a schematic of the approach and the datasets used for model generation, validation and verification.

To reduce the impact of data noise on model performance, we first reduced the dimensionality of the omics datasets by obtaining empirical markers of drug responses (EMDRs, Fig. 1a, c), which are used to compute an overall metric of drug response distance (*D*). EMDR were identified using 80% of the samples in the training set. The *D* metric is the difference in overall expression of markers increased in drug sensitive cells relative to markers increased in drug resistant cells within a sample. This is an important feature of DRUML for two reasons: firstly, the use of averaged marker values circumvents the problem of missing predictors when making predictions in verification or in future datasets because *D* can be computed even in cases where omics input data have missing values; secondly, *D* is an internally normalized metric obtained by subtracting averaged signals from two sets of phosphosites, proteins or transcripts within a given sample; therefore, once the model is built, application of DRUML to predict drug responses in a new cancer-derived sample does not require comparison against a control or reference sample set.

### Input datasets

To develop DRUML, we first analyzed the proteomes and phosphoproteomes of a panel of 26 AML, 10 esophageal and 12 hepatocellular carcinoma cell lines in triplicate (three independent cultures per cell line) by LC-MS/MS as described previously^25,36^ (Fig. 1b, details are given in Supplementary Data 1). This analysis required 288 LC-MS/MS runs and produced a sufficiently large basal phosphoproteomics and proteomics dataset containing 22,804 phosphopeptides and 6455 proteins, which generated 3,283,776 and 929,520 quantitative data-points, respectively (Supplementary Fig. 2a). Unsupervised hierarchical clustering showed that AML cell lines separated from those obtained from solid tumors and individual replicates grouped together (Supplementary Fig. 2b), thus highlighting the quality of the quantitative data. We provide these large datasets of phosphoproteomics with matched proteomics data for AML, esophageal and hepatocellular carcinoma cell lines available as a community resource (Supplementary Data 2 and 3).

Drug response data in the form of area above the curve (AAC values) were obtained from PharmacoDB^32^ for the same cell lines for which we produced phospho- and proteomics data. To normalize for essay conditions, AAC values were scaled so that values ranged from 0 (no effect of drug) to 1 (maximum cell killing) within a given cell line. Drugs were filtered by an interquartile range > 0.15 AAC units to ensure that there was a sufficient range of sensitivity, thus reducing the number of profiled drugs from 659 to 466. For comparison we also used RNA-seq data obtained from the DepMap portal^37^ as the input of the models. Supplementary Fig. 2c shows that principal component analyses of the proteomics, phosphoproteomics and drug response data grouped cell lines by cancer type. Thus, to ensure that the models generated were interrogating the biological mechanisms of sensitivity without the influence of tumor type, we constructed separate DRUML models for solid and AML tumor samples as explained below.

### Dimensionality reduction

To illustrate the approach that we used to reduce dimensionality, Fig. 1c-f show the determination of EMDRs for BYL-719 (alpelisib), a small molecule inhibitor with selectivity for Class Ia PI3Kα^38^. We employed a tenfold cross validation method, in which for each drug, 80% of cell line samples (the training set) were split into those that are resistant or sensitive (using the median AAC cutoff) to each particular drug. These sensitive and resistant groups were further split into tenfolds each and proteins, phosphosites and transcripts were compared (by Limma, Fig. 1c) by repeated resampling in the resistant vs sensitive folds showing significantly different drug response values. Markers consistently found to be increased or decreased in sensitive cells are stored as EMDRs and provided in the DRUMLR package (Supplementary Data 4). We refer to markers increased in sensitive cells as sensitivity markers and those decreased as resistance markers. As outlined above, our approach then involves combining the identified EMDRs into a distance metric (*D*), which is essentially a measure of the distributions of sensitivity markers relative to resistance markers; *D* is formally defined in Fig. 1a. Figure 1e shows the distribution of phosphorylation site markers associated to resistance and sensitivity to BYL-719. These distributions are then measured to derive *D* values (Fig. 1e), which correlate with drug responses across these cell lines and across all models tested (Supplementary Fig. 3a–c). As expected, the correlation was statistically significant when markers derived from AML or solid tumors were applied to the respective tumor type but not when solid tumor derived markers were used to compare AML cell responses and vice-versa (Supplementary Fig. 3a–c).

**Fig. 2:**
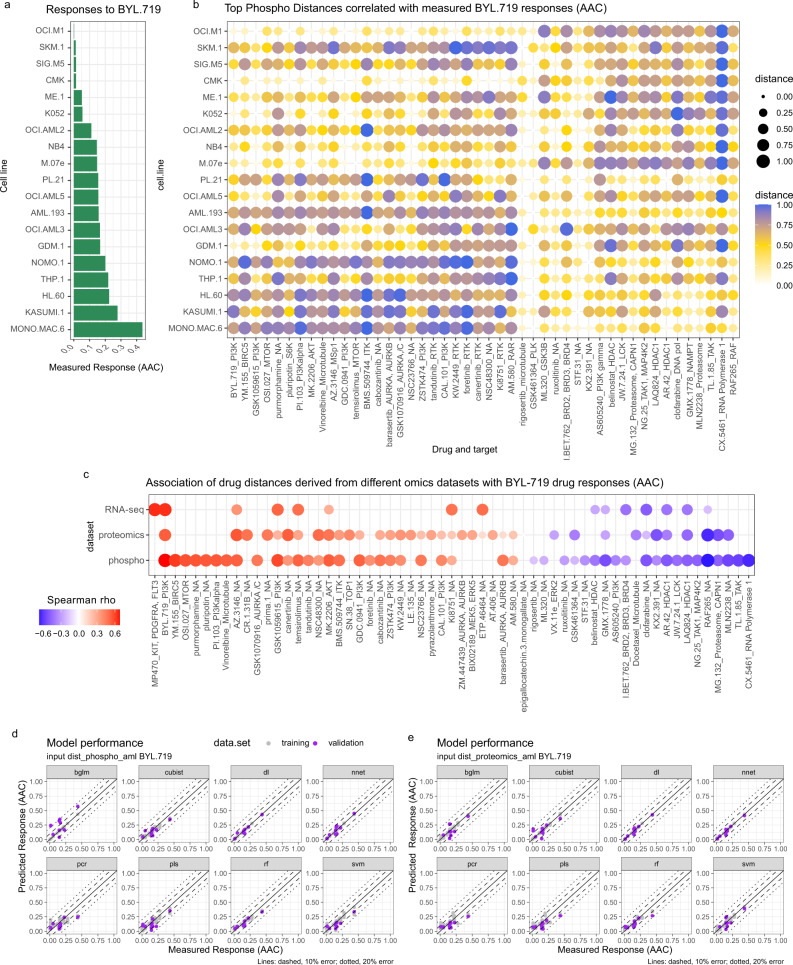
Dimensionality reduction using empirical markers of drug responses. **a** Barchart of average AML cell line sensitivity to BYL-719 (*n* = 19) rows are sorted in order of BYL-719 sensitivity (AAC). **b** Mean expression of top drug marker distance (*D*) values which correlate both positively and negatively with drug sensitivity to BYL-719 (2 tailed spearman correlation coeffiecient) in AML omics datasets (*n* = 19), measured in triplicate. Rows are sorted in order of BYL-719 sensitivity (AAC). Dot color intensities and sizes are proportional to distance values normalized 0–1. Rows in heatmap are ranked based on BYL-719 sensitivity; columns are ordered based on hierarchical clustering (complete with Euclidean distance). **c** Overall correlation of each distance marker with BYL-719 sensitivity; red values correlate with sensitivity whilst blue values correlate with resistance. Dot sizes are proportional to Spearman rho value. Columns in heatmap are ordered based on hierarchical clustering (complete with Euclidean distance). **d** Comparison of measured vs predicted responses retuned by the eight different learning methods using phosphoproteomics data as input. Solid, dashed and dotted lines signify 0%, 10% and 20% absolute error boundaries, respectively. **e** As in (**d**) but *D* values were obtained from proteomics data. Learning algorithms were random forest (rf), cubist, bayesian estimation of generalized linear models (bglm), partial least squares (pls), principal component regression (pcr), support vector machine (svm), deep learning (dl) and neural network (nnet).

To reduce dimensionality further, for each drug, DRUML selects the top *D* values positively and negatively correlated with the dose-response data across the cell lines in the training set for all the 466 drugs. Supplementary Fig. 3d and 3e show that BYL-719 responses are correlated with *D* values for several drugs. As a positive control for the approach, we found that BYL-719 *D* values obtained from the three different marker datasets were consistently correlated with responses to this drug (Fig. 2a–c and Supplementary Fig. 3a–c). Figure 2a, b depicts the distance values of several drugs for cell lines ranked based on their response to BYL-719. Figure 2c shows Spearman rho values of the association between responses to BYL-719 and *D* values obtained from transcriptomic, proteomic and phosphoproteomic datasets. This analysis illustrates that *D* values were similarly correlated with BYL-719 responses across the different omic datasets. In addition to BYL-719 *D* values, responses to this drug were also correlated with *D* values for other Class Ia PI3K inhibitors (GSK1059615, PI-103, GDC-0941 and ZSTK474), and with *D* values for inhibitors of kinases acting downstream and upstream of PI3K, including of mTOR (tensirolimus and OSI-027), AKT (MK-2206), SK6 (pluripotin) and receptor tyrosine kinases. BYL-719 responses were also correlated with *D* values for Aurora A/B inhibitors and were anti-correlated with *D* values for inhibitors against several HDAC isoforms (including AR-42, belinostat, and LAQ824) and RAF kinase (FAR265) among others (Fig. 2a–c and Supplementary Fig. 3d–e). The anti-correlation between responses to PI3K and HDAC inhibitors is consistent with these two inhibitor classes being synergistic in decreasing cancer cell viability^39^. Thus, *D* values from different drugs and datasets produced biologically meaningful results and reproducibility correlated with drug responses irrespective of the omic datasets from which these were obtained.

### ML models of responses to BYL-719

We next generated predictive ML models of drug responses using the top correlated *D* values for a given drug obtained as explained above for BYL-719 (Figs. 1 and 2 and Supplementary Fig. 1). Since we did not have prior knowledge of the learning methods that would be more appropriate to predict drug responses from our datasets, we first assessed the performance of diverse ML methods based on random forest (rf), cubist, bayesian estimation of generalized linear models (bglm), partial least squares (pls), principal component regression (pcr), support vector machine (svm), deep learning (dl) and neural network (nnet) learning algorithms. Although, as discussed above, our primary aim was to compare models constructed from *D* values obtained from phosphoproteomics and proteomics data, for benchmarking, we also constructed models using *D* values obtained from RNA-seq data as the input. The RNA-seq dataset was obtained from public resources^18^ making it difficult to directly compare the results obtained using RNA-seq with those obtained from our in-house generated proteomics and phosphoproteomics data. Therefore, in this study we do not derive conclusions on the relative performance of RNA-seq derived models. For model generation, samples from the training set were used to train regression models on the normalized drug response (AAC) data by tenfold cross validation using the root mean standard error (RMSE) metric as the loss function. DL/ML models were then evaluated on the validation set by comparing predicted vs actual responses using absolute error or standard error (SE) and RMSE (for individual data points and overall model performance, respectively).

Using BYL-719 as an initial example, we assessed the performance of the different models generated using *D* values derived from phosphoproteomic and proteomic datasets as predictors (shown in Fig. 2d). Figure 2d, e show that DL and NNET using D values from phosphoproteomics data produced models with the smaller validation errors across all cell lines (absolute errors < 0.2 AAC units, Fig. 2d, e). DL models were able to predict 12 out of 12 validation data-points within <0.1 AAC units.

### Systematic identification of EMDRs and ML model generation

**Fig. 3:**
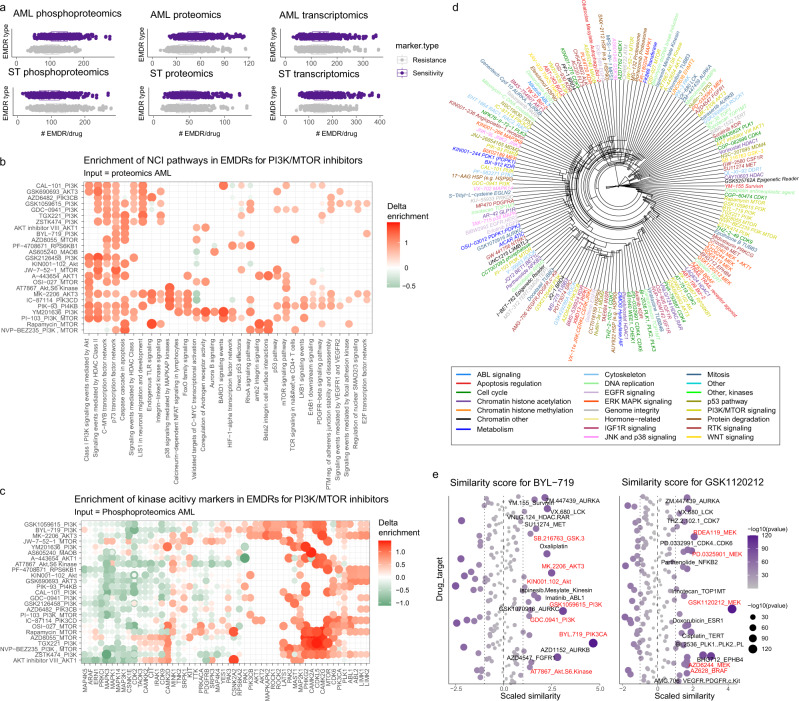
Overview of systematic empirical markers of responses for >400 drugs. **a** Total number of empirical sensitivity and resistance markers identified per drug in AML omics data (*n* = 26, measured in triplicate). Boxplots have median centers, interquartile box boundaries and range upper and lower hinges. Not all drugs were profiled in all cells cell lines; markers were successfully identified for 466 drugs with sufficient data points. **b**, **c** Mean enrichment of NCI pathways (**b**) and kinase activity markers (**c**) in EMDRs for PI3K/MTOR pathway inhibitors. Rows and columns in heatmap are ordered based on hierarchical clustering using Euclidean distances. Delta enrichment was calculated as the enrichment of the named pathway or kinase substrate set in EMDR increased in sensitive cells to a given drug minus the enrichment of the same pathways or kinase substrate in EMDRs increased in resistant cells. **d** Unsupervised clustering of delta enrichment values for all drugs with known targets (hierarchical clustering using Euclidean distances). **e** Drugs similar to BYL-719 and GSK1120212 (trametinib) as determined by similarity scores calculated from the Pearson correlation of pathway enrichment values between drug pairs. Pearson values were scaled and *p* values determined by comparing each similarity score value against the distribution of all values using one sample *t*-test.

Systematic application of this approach (Fig. 1c, Supplementary Fig. 1) to 466 drugs in AML and solid cancer cell lines (Fig. 3a) led to the identification of 1232 and 1139 phosphorylation sites, 542 and 480 proteins and 3046 and 3699 transcripts markers of responses for AML and solid models, respectively. On average, each drug was annotated with 128 ± 37 (mean ± SD, range 53–278) and 97.6 ± 43 (15–269) phosphorylation sites markers of drug responses for AML and solid models respectively, and with 40–50 protein markers of resistance or sensitivity on average in solid and AML models (10–131 range) (Supplementary Fig. 4). The number of RNA transcripts associated to drug responses was greater because of the size of the input data. As Supplementary Fig. 5a-d illustrates, several of these phosphorylation sites, proteins and transcripts were found to be markers of responses for several drugs. Of interest, phosphorylation sites on FAM129B, SRRM2, lamin (LMNA) and on the mTOR substrate 4EBP1 were found to be sensitivity markers for >200 drugs, whilst NPM1 (protein name nucleophosmin, NPM) phosphosites and the full protein were frequent markers of resistance (Supplementary Fig. 5c).

We also sought to explore the biological relevance of our EMDRs. We hypothesized that these proteins and phosphorylation sites should show commonalities between drugs with similar modes of action, and thus group together by EMDR similarity. To test this idea, we performed a systematic ontology, pathway and kinase substrate enrichment analysis of the EMDRs (Supplementary Data 5). For each drug and pathway, a delta enrichment value was calculated as the difference between enrichment of sensitivity and resistance EMDRs for such drug and pathway. The results of these analysis, provided in Supplementary Data 5, allow exploring drugs’ modes of actions systematically. Examples of this analysis in Fig. 3a, b show the enrichment of NCI pathways and kinase downstream targets in EMDRs of PI3K/MTOR/AKT pathway inhibitors. Pathways such as “Class I PI3K signaling events mediated by Akt” and “p73 transcription factor network” were enriched in EMDR for this drug class (Fig. 3b). Kinase activity readouts enriched in sensitivity markers for PI3K/MTOR/AKT pathway inhibitors included, among others, those for PIK3CA and MTOR, whereas kinase targets for several other kinases, such as MAPK1/3 and PCKI, were enriched in resistance markers (Fig. 3c). These data are consistent with the role of MAPK and PKC pathways in compensating for PI3K inhibition is some settings^40^. Kinase inhibitors are notoriously promiscuous^41^. However, despite these compounds having different off-target effects, unsupervised hierarchical clustering of the delta enrichment values grouped 7 out of the 17 PI3K/MTOR inhibitors together (Fig. 3d). Similar effect was observed for MEK inhibitors (4 out of 6 clustered). To investigate whether our dataset may be explored to identify drugs with similar mode of action, we correlated the pathway/ontology delta enrichment values for all drugs. We then calculated similarity scores between drug pairs as the Pearson correlation coefficient of the pathway enrichment values for such pair of drugs (results are provided in Supplementary Data 6). To illustrate this analysis, Fig. 3e shows that the most similar drug to the PI3K inhibitor BYL-719 was found to be GSK1059615, a compound that also has PI3K as the intended target. Similarly, similar drugs to the MEK inhibitor GSK1120212 (trametinib) included the MEK inhibitors RDEA119 and PD.0325901) as well as BHG712 (which targets EPHB4, a cell surface receptor upstream of MEK^42^) and the PLK1 inhibitor BI-2536, consistent with the potential of PLK1 in regulating MEK/ERK pathway activity^43^. As a further example, drugs similar to the MTOR inhibitor rapamycin (as judged by similarity score analysis) included several PI3K, ATK, S6K and CDK1 inhibitors (Supplementary Fig. 5e). Thus, our markers of drug response are on the whole consistent with the drugs’ mode of action, thus suggesting that these markers are indicative of the biological mechanisms that determine responses to the profiled drugs.

### DRUML model ensembles to rank drug responses

**Fig. 4:**
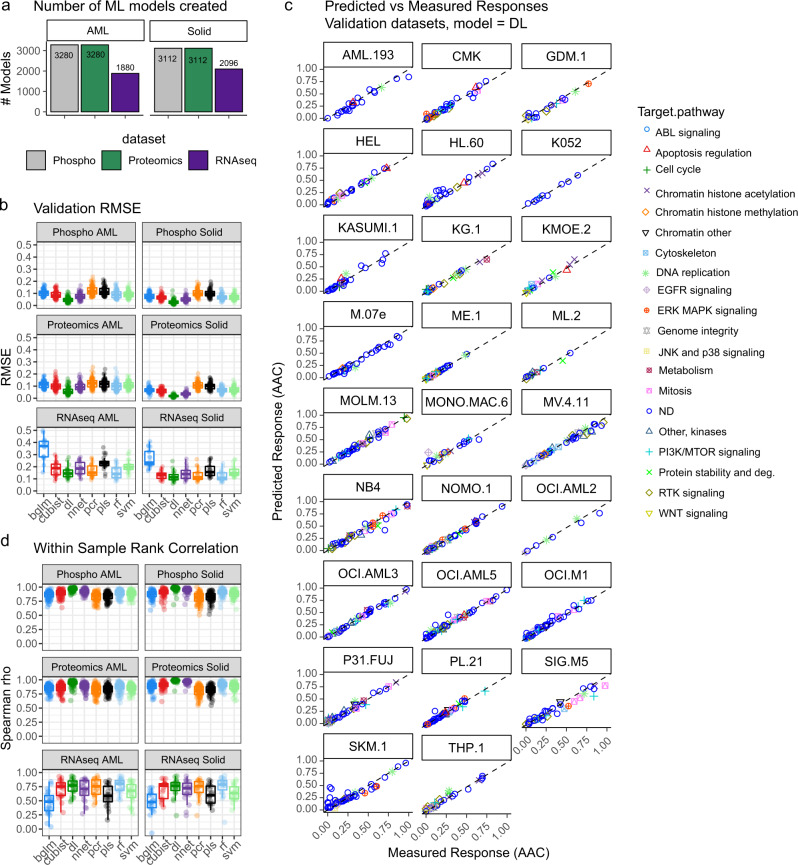
Performance and accuracy of DRUML to rank drugs based on efficacy. **a** Total number of models generated from phosphoproteomics, proteomics and RNA-seq data. Input data were split into solid (*n* = 22) and AML (*n* = 26) cell line groups for model building. **b** Validation errors for each model generated binned by ML method and input dataset. **c** Comparison between measured and predicted drug response values produced by DL analysis of phosphoproteomics distance values in the AML datasets. Each data-point represents a drug prediction color coded by the drugs’ mode of action. Each cell line was analyzed in triplicate and representative comparisons are shown. Dotted line denotes slope of 1 with 0 intercept. **d** Spearman rank correlation coefficient rho values between predicted and actual responses returned by the different learning models and input datasets). Boxplots (**b** and **d**) have median centers, upper and lower quartile box boundaries and range upper and lower hinges. Learning algorithms were random forest (rf), cubist, bayesian estimation of generalized linear models (bglm), partial least squares (pls), principal component regression (pcr), support vector machine (svm), deep learning (dl) and neural network (nnet).

Next, we used the approach described above (using BYL-719 as an example) to systematically construct predictive models for 466 drugs (of which 412 could be modeled) using the phosphoproteomics, proteomics and RNA-seq distance *D* data obtained from AML and solid tumors as input. In total we constructed 16,760 learning models (Fig. 4a). About the same number of models were created from phosphoproteomic and proteomics datasets (Fig. 4a). As with the analysis of models of BYL-719 sensitivity, the DL algorithm produced the smaller validation errors for the proteomics and phosphoproteomic data derived from solid and AML tumor cancer types with RMSE < 0.1 in all cases (Fig. 4b).

We then tested whether the ML models would allow ranking drugs within a cell line based on their predicted efficacy. Figure 4c shows the ranking of drugs in AML cells used for the validation of the DL algorithm. We observed a remarkably high correlation between the predicted and actual responses within cell models across drugs of diverse mode of action. The RMSE between predicted and actual responses were 0.078, 0.040 and 0.13 for DL models derived from phosphoproteomics, proteomics and RNA-seq datasets, respectively (Fig. 4b). Spearman rank correlation analysis confirmed the existence of a strong correlation between experimental and modeled drug responses (with mean Spearman rho = 0.88, *q* values < 0.002., Fig. 4d, Supplementary Data 7). Thus our results suggest that DRUML may be used to accurately rank drugs of diverse mode of action within tumors based on their predicted efficacy.

### Verification in independent datasets

Ultimately, to be useful, predictive models of drug response should be able to accurately predict treatment outcome irrespective of the laboratory from which the data was obtained. Therefore, to verify DRUML using data collected by independent laboratories, we strived to test whether the models generated with our training datasets would predict drug responses from publicly available label-free proteomics and phosphoproteomics datasets generated by other groups. Label-free phosphoproteomics data from 8 colorectal cancer cell lines from Piersma et al. was sourced from PRIDE [^44^, pride id: PXD001550] and reprocessed using an in house mass spectrometry informatics pipeline^28,36^, leading to the identification and label-free quantification of 12,197 phosphopeptides (Supplementary Fig. 6). This dataset was then used as an input for DRUML to predict drug responses from the models previously generated using solid tumor’s phosphoproteomics data for six of these cell lines (for which drug response data is available). To do this, we generated drug *D* values (using the EMDR obtained from previous work (Fig. 3) from this new dataset as outlined above (Supplementary Fig. 1 and Methods), which we then used as the input of our saved DRUML models (created using esophageal and liver cancer phosphoproteomes). This analysis led to the prediction of responses for 389 drugs (number of profiled drugs varied across individual cell lines), which were then compared with experimental data for drugs and cell lines present in drug response repositories.

**Fig. 5:**
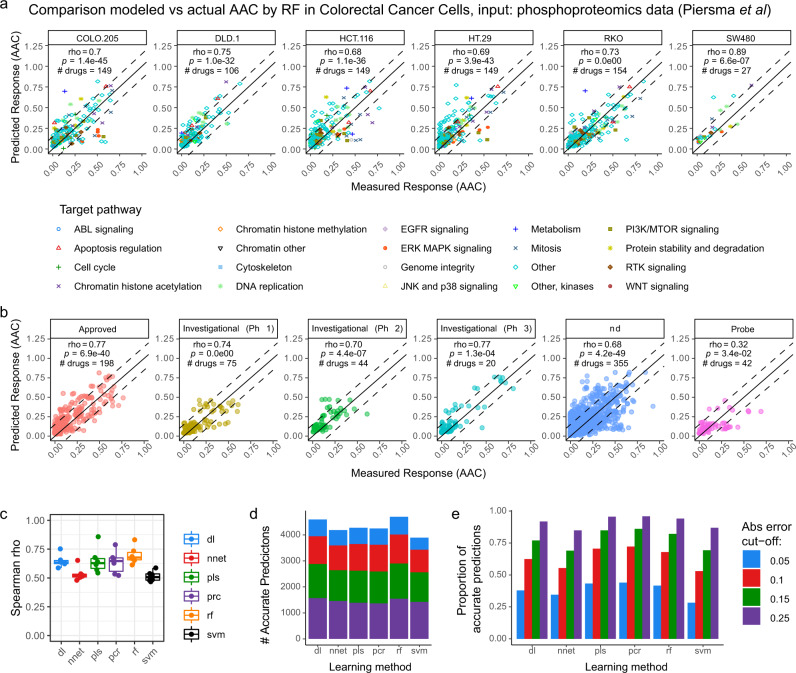
Accuracy assessment of DRUML to rank drugs based on efficacy using an independent phosphoproteomics dataset. DRUML was used to predict responses to 389 drugs in the colorectal cancer (CRC) cell lines shown using phosphoproteomic data obtained from Piersma et al (Jimenez lab, PRIDE PXD001550) **a**, **b** Scatter plots of measured and predicted drug responses. Dotted lines represent 10% absolute error margin, each data-point represents a drug prediction and statistical significance of drug ranking was assessed by measuring Spearman’s rank correlation coefficient. **a** Model performance across cell lines (*n* = 8). Drugs are colored by mode of action. **b** Comparison of measured and predicted drug responses binned by developmental stage of the drug. **c** Distribution of Spearman rho values by ML model. Boxplots represent median centers, interquartile box boundaries and range upper and lower hinges. **d**, **e** Number (**d**) and proportion (**e**) of accurate predictions of drugs for all cell lines within 0.05, 0.1, 0.15 and 0.25 absolute errors. Learning algorithms were random forest (rf), cubist, bayesian estimation of generalized linear models (bglm), partial least squares (pls), principal component regression (pcr), support vector machine (svm), deep learning (dl) and neural network (nnet).

We observed a significant correlation between the DRUML-derived drug response predictions and the actual responses for these six cell lines across drugs with diverse mode of action (Fig. 5a) and developmental phase (Fig. 5b). Associations were statistically significant with *p* values (by Spearman Rank correlation) ranging from *p* = 2.1e-06 to *p* = 1.4e-45 (Supplementary Data 8). In this dataset, PCR and RF learning algorithms performed as well or better than DL. For the RF models, Spearman rho was 0.70 ± 0.077 (mean ± SD, *n* = 6 cell lines) (Fig. 5c) and mean *q* value = 1.1e-07 with >85% of all responses being predicted with absolute errors <0.15 AAC units (Fig. 5d, e). We also compared the predicted vs observed rankings of drug responses within given cancer cell lines for all drugs (Supplementary Fig. 6c) and for the top 20 ranked drugs (Supplementary Fig. 6d). The best performing learning algorithm was RF, with which 88% of drug responses were predicted with differences in ranking (predicted vs observed) < 50 positons and 86% of them with raking differences < 20 positions. For the top 20 ranked drugs, 51% of predictions had a difference in ranking < 50 positons and for 45% of them the difference in raking prediction was < 20 positions.

**Fig. 6:**
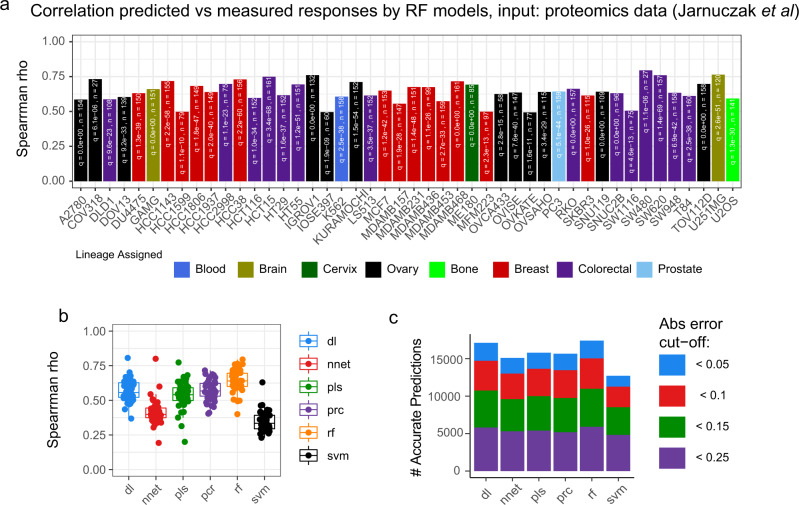
Accuracy assessment of DRUML to rank drugs based on efficacy using independent proteomics datasets derived from 47 tumor models and 8 pathologies. DRUML was used to predict drug responses for 47 cell lines using proteomic data obtained from 12 different laboratories and compiled by Jarnuczak et al. (Vizcaino lab, PRIDE PXD013455). Statistical significance of predictions was measured using Spearman’s rank correlation coefficient and Rho values are shown in figures (**a** and **b**). **a** Comparison of measured vs predicted drug responses within cellular models using the random forest method. Samples are colored by sample origin. **b** Distribution of Spearman rho correlation values between predicted and actual drug responses for all samples separated by ML learning algorithm. This boxplot has median centers, interquartile box boundaries and range upper and lower hinges. **c** Comparisons of the proportion of accurate predictions at 0.05, 0.1, 0.15 and 0.25 error cut-offs returned by the different ML methods. Learning algorithms were random forest (rf), cubist, bayesian estimation of generalized linear models (bglm), partial least squares (pls), principal component regression (pcr), support vector machine (svm), deep learning (dl) and neural network (nnet).

We next applied DRUML to predict drug responses in a panel of 47 cell lines derived from diverse solid tumor types. The input for this analysis was proteomics data obtained from Jarnuczak et al. [^45^, pride id: PXD013455], who compiled data from 11 separate studies. As with the analysis of the CRC dataset, our set of EMDRs were used as input of DRUML models—in this case generated with our solid tumor proteomics training dataset (Figs. 1 and 3, Supplementary Fig. 1)—to predict responses from iBAQ values provided by Jarnuczak et al. without further processing (except for the averaging of iBAQ values of replicate cell line measurements). Figure 6a and Supplementary Fig. 6 show that DRUML-predicted and actual drug responses were highly associated across the 47 cell lines irrespective of their assigned pathology as assessed by Spearman correlation of predictions generated by the RF models (rho values were 0.64 ± 0.83 (mean ± SD) with *p* values < 1e-05 for all cell line comparisons, values are provided in Supplementary Data 9). Mean square error (MSE) of prediction was below 0.1 for all cell lines (Supplementary Fig. 7a, b). Models generated by RF showed the greater Spearman rho values of association between predicted and measured responses within cell lines (Fig. 6b), and as with the predictions from phosphoproteomics data, >85% of drug responses were predicted with absolute errors <0.15 AAC units, and 95% of them with errors <0.25 AAC units, with RF model producing the most accurate predictions (Fig. 6c). Similarly, for 51% of predictions based on RF, the differences in drug response ranking (predicted vs measured) within given cancer cell lines were <20 ranking positions (Supplementary Fig. 7c); for the top 20 ranked drugs per cell model, 76% of predictions showed rankings differences of <20 positions (Supplementary Fig. 7d). Overall, these data indicate DRUML to accurately predict and rank the efficacy of drugs of diverse mode of action in cancer cells derived from different pathologies using proteomics data obtained using routine LC-MS/MS from different laboratories.

### Assessing DRUML in a clinically relevant sample set

**Fig. 7:**
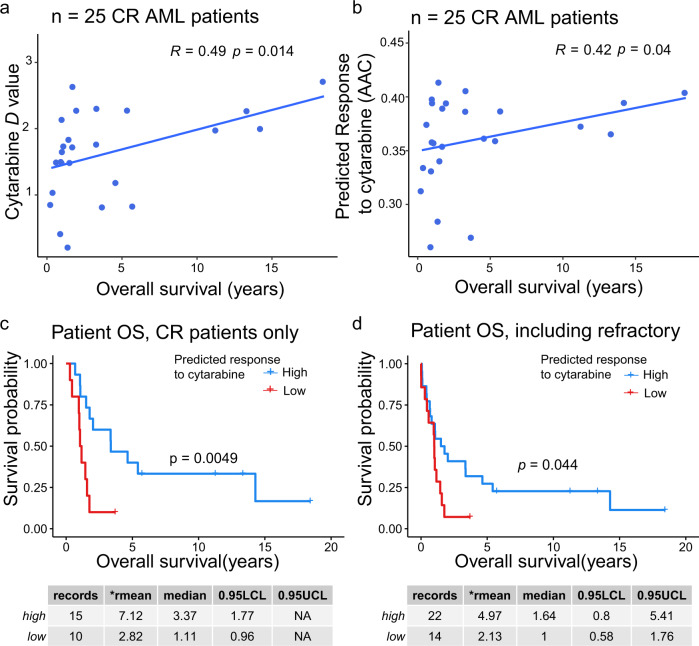
Cytarabine DRUML model predicts prognosis of AML patients treated with cytarabine. DRUML was used to predict responses to cytarabine from phosphoproteomics data obtained from 36 AML patients in triplicate by Casado et al. (PRIDE PXD005978). Prediction was obtained from the average prediction derived from random forest, principal component regression and partial least squares models. **a**, **b** Correlation between overall patient survival and cytarabine *D* values (**a**) or DRUML predicted responses (**b**) in patients that underwent complete remission (CR) and received consolidation therapy (*p* values by Spearman, *n* = 25). **c**, **d** Kaplan–Meier survival curves of patients with high (blue lines) or low (red lines) DRUML predicted cytarabine responses for CR (**c**) and for all (**d**) patients (*p* values by log-rank test). Mean of predicted cytarabine AAC was used as cut-off for patient stratification.

To assess whether DRUML-derived drug efficacy prediction may be clinically relevant, we predicted responses of AML patients to cytarabine in a published AML phosphoproteomics sample set obtained from Casado et al. containing data for 36 primary AML samples^25^. Patients in this cohort were treated with induction chemotherapy consisting of cytarabine complemented with co-treatments with an anthracycline (daunorubicin or doxorubicin). AML patients that achieve complete remission (CR) undergo consolidation therapy with low dose cytarabine^46^. Therefore, we reasoned that AML patients predicted to be sensitive to cytarabine by DRUML would show greater clinical overall survival (OS) than those predicted to be resistant. *D* values were calculated from the analysis of EMDR in this primary AML phosphoproteomic dataset, and these were used as input of saved DRUML models to predict cytarabine responses. Patient OS was significantly correlated with the *D* values for cytarabine (Spearman *p* = 0.014, Fig. 7a) and with predicted responses to this drug (Spearman *p* = 0.04, Fig. 7b). Similarly, OS analysis using the Kaplan–Meier method showed that patients with high predicted cytarabine responses (greater than the mean predicted AAC) survived longer than those with low predicted responses (Fig. 7c, d). Median OS was 1.1 vs 3.4 years (*p* = 0.0049, *n* = 10 and 15 respectively) for patients that underwent CR, whereas in the complete sample cohort median OS was 1.0 and 1.64 years for low and high cytarabine predicted response groups, respectively (*p* = 0.044). These results are consistent with the fact that patients who achieve CR are treated with cytarabine in consolidation therapy regimes, whereas refractory patients do not receive this treatment^46^. These results therefore indicate that DRUML-predicted drug responses are clinically relevant in this setting.

## Discussion

In this study we have demonstrated that large scale proteomics and phosphoproteomics data can be used as an input for DL/ML to rank drugs based on their predicted anti-proliferative effects in a given cancer cell population. To facilitate this work, we developed DRUML, an ensemble of predictive models trained for 412 drugs with different mode of action and developmental stage. This study was possible because methods for systematic and relatively high-throughput label-free analysis of proteomes and phosphoproteomes are now emerging^24,28,31,47–51^, and drug response data for a large number of compounds have been made public recently^18,32,34,35^. However, since the use of large-scale LC-MS/MS proteomics data for DL/ML model generation has not been investigated systematically before, as part of DRUML development, we assessed the suitability of such large scale datasets as the input of predictive drug response models. In contrast to small scale proteomic studies based on protein array methods^52,53^, which measure a few dozens of proteins and phosphorylation sites, our study was based on the analysis of >20,000 phosphorylation sites and ~7000 proteins, thus allowing for systematic and unbiased discovery of drug response markers. Our initial evaluation showed that phosphoproteomics data consistently produced the lowest training and validation errors, although the difference between the accuracy of proteomics- and phosphoproteomics-based models was small. This is consistent with previous findings from our and other laboratories, which found that phosphoproteomics and proteomics data reflect the mechanisms underpinning drug responses of cancer cells^22,24–27^.

To limit the impact that noise and missing values in omics datasets may have in DL/ML model performance, and to make the approach practical, instead of individual phosphosites, proteins or transcripts, DRUML uses as input a distance metric (which we termed *D*) that measures the differences in distribution levels between sensitivity and resistance markers for a given drug. This feature contributes to the robustness of DRUML as *D* is an internally normalized value which uses biologically relevant selected markers, thereby reducing data noise and addressing the potential issue of missing features in validation and verification datasets. Indeed, since each *D* value is calculated on average by hundreds of proteins, phosphorylation sites or transcripts (Fig. 3, Supplementary Fig. 4), *D* metrics may be computed even when there are missing EMDRs in the datasets being analyzed, bypassing the need for imputation. DRUML uses *D* values for the respective drug and those calculated for other drugs. For example, the *D* values chosen to build DL/ML models for BYL-719 include BYL-719 *D* values and also *D* values for inhibitors of enzymes in pathways that act in parallel, upstream and downstream of PI3K (BYL-719 main target, Fig. 2). Thus, consistent with BYL-719 known mode of action, selected *D* values for BYL-719 ML model generation, consisted of those for PI3K, AKT, mTOR and RTK inhibitors (which positively correlated with responses to BYL-719) but also *D* values for drugs against HDAC, which are known to synergize with PI3K inhibitors^39^. This finding, together with the observation that our EMDR sets grouped drugs based on their mode of action when analyzed by unsupervised classification methods (Fig. 3), suggest that EMDRs, used to calculate *D* values for DRUML model generation, reflect the biological mechanisms of responses to the different drugs. We believe that the biological relevance of the composite input features contributed to the models’ predictive accuracy in independent datasets (Figs. 5, 6, 7) despite these being trained with a relatively small number of samples.

In our study, to avoid overfitting, we limited the number of *D* values to construct DL/ML models for each drug to a maximum of 60 (with a minimum of 14). By controlling the number of *D* values to be included in models, it is possible to optimize learning model performance. The calculation of *D* values relies on measuring EMDR and we obtained >2000 phosphorylation sites and >800 proteins of such EMDR as input for DRUML; these are made available to the community (Supplementary Information).

Previous studies have suggested that, in general, drug efflux pump expression is the predominant variable impacting drug resistance for a variety of drugs^27^ and that, in particular, kinase activation (detected as phosphorylation of kinase activity markers) underlies responses to kinase inhibitors^24,25^. In our work we found pathways, ontologies and kinase substrates enriched in sensitivity and resistance markers for the 466 drugs for which we obtained EMDR (Fig. 3, Supplementary Data 5). In general drugs targeting specific pathways had EMDR enriched in similar pathways thus reflecting their mode of action. These data, provided in Supplementary Data 5 and 6, allow exploring drugs’ mechanisms of responses and their similarity with other drugs.

A limitation of DRUML is that the drugs for which responses may be predicted are limited to those present in current repositories of drug responses. In this work we were able to predict the ranking of 412 drugs within a cancer cell line but many of those compounds are probes, which are unlikely to progress into the clinic. As new drugs are developed by the pharmaceutical industry, these could be incorporated into a retrained DRUML model that captures all clinically relevant drugs. DRUML was developed using data obtained from cancer cell lines, which although they recapitulate some of the biology of the tissue from which they originate, they have gone through the process of immortalization and their growth conditions are different from those in vivo. It is therefore arguable whether the mechanisms by which primary tumors respond to drugs are preserved in immortalized cell lines. Notwithstanding these considerations, studies have shown that mechanisms of drug resistance identified in cell lines can be clinically relevant^54^. Similarly, we observed that the cytarabine DRUML model predicted OS of AML patients treated with this drug (Fig. 7), suggesting that, in at least some cases, DRUML models may be able to predict clinical drug responses.

Out of the different learning algorithms tested, we found that PCR and RF produced lower errors in verification datasets obtained from independent laboratories (Figs. 5 and 6), while the DL models exhibited strong overtraining. This contrasts with findings from previous studies using transcriptomic data as input, which found that DL outperformed other ML methods^55^. This difference may be explained by the fact that DL performance is only significantly greater than that provided by other ML methods when applied to large datasets^56^. Therefore, DL methods may be better suited to train predictive models from phosphoproteomics and proteomics as larger data sets become available.

Assessment of DRUML using external verification datasets from 53 cell lines analyzed by independent laboratories^44,45^ revealed that around 85% of the drugs could be ranked with absolute errors < 0.15 and the drug rankings were statistically significant (by Spearman) within all cancer models tested (Figs. 5 and 6). This represents a remarkable and surprising accuracy given that DRUML was trained using esophageal and liver cancers, whereas the verification datasets consisted of data from cell lines derived from bone, brain, breast, cervix, colorectal, ovary and prostate cancers.

In summary, in this study we have assessed the accuracy of DRUML to produce list of drugs ranked by their predicted efficacy in reducing the proliferation of a given cancer cell population. We trained and validated the approach with the analysis of 48 cell lines profiled in our laboratory and verified it with a set of 53 cancer cell models profiled by twelve other groups and in a 36 primary AML sample set. Our results indicate that DRUML ranks drugs of different mode of action based on their predicted efficacy across different cancer types with reasonable low error. Ultimately, DRUML could assist drug prioritization by complementing information obtained from clinicopathological parameters and mutational analysis.

## Methods

### AML cell lines

The AML cell lines AML-193, CMK, K-052, Kasumi-1, KG-1, HEL, ME-1, ML-2, MOLM-13, MONO-MAC-6, MV4-11, OCI-AML2, OCI-AML3, OCI-AML5, P31/FUJ, PL-21, SIG-M5, SKM-1 and THP-1 were derived from male patients while, GMD-1, KMOE-2, HL-60, M-07e, NB-4 and NOMO-1 were derived from female patients. The sex of the patient from which OCI-M1 cells were derived is not specified in the DSMZ-German Collection of Microorganisms and Cell Cultures GmbH database.

Briefly, SIG-M5 and M-07e cell lines were maintained in IMDM supplemented with 20% FBS and 1% Penicillin/Streptomycin (P/S). M-07e cells were in addition supplemented with 10 ng/mL of GM-CSF. OCI-M1 and AML-193 cells were grown in IMDM supplemented with 10% FBS and 1% P/S. AML-193 cells were in addition supplemented with 5 ng/mL of GM-CSF. OCI-AML2, OCI-AML3 and OCI-AML5 cell lines were grown in α-MEM supplemented with 20% FBS and 1% P/S. OCI-AML5 cells were also supplemented with 5 ng/mL of GM-CSF. K-052 cells were maintained in α-MEM supplemented with 10% FBS and 1% P/S. GDM-1 and SKM-1 cells were grown in RPMI-1640 supplemented with 20% FBS and 1% P/S. SKM-1 cells were also supplemented with 1 ng/mL of GM-CSF. All other AML cell lines were maintained in RPMI-1640 supplemented with 10% heat inactivated FBS and 1% (RPMI/FBS medium). All cell lines were maintained at 37 °C and 5% CO_2_ in a humidified environment. Cell density was kept between 0.5 and 1.5 × 10^6^ cells per mL.

For proteomics and phosphoproteomics analysis, AML cell lines were seeded in IMDM medium supplemented with 10% FBS and 1% P/S in T75 flask (20 × 10^6^ cells in 10 mL) and incubated for 3 h at 37 °C and 5% CO_2_ in a humidified environment. Cell suspensions were then transferred to 15 mL falcon tubes and centrifuged at 520 × *g* for 5 min at 5 °C. Supernatants were removed and cell pellets were washed twice with ice cold DPBS supplemented with phosphatase inhibitors (1 mM NaF, 1 mM Na_3_VO_4_). During washes, cells were centrifuged at 520 × *g* for 5 min at 5 °C. Cell pellets were transferred into 1.5 mL protein LoBind tubes, snap frozen in dry ice and stored at −80 °C. Biological independent replicates for each cell line (*n* = 3) were performed in different dates.

### Hepatic cancer cell lines

The hepatic cancer cell lines HEP 3B2.1-7, HEP G2, JHH2, JHH4, SK-HEP-1, SNU182, SNU-398, SNU-423, SNU-449 and SNU-475 were derived from male patients while the cell line SNU-387 was derived from a female patient. The gender of the patient from which PLC/PRF/5 cells are derived is not specified in the American Type Culture Collection database.

Cell lines SNU-387, SNU-423, SNU-182, SNU-398, SNU-475 and SNU-449 were maintained in RPMI-1640 supplemented with 1 mM sodium pyruvate, 10% FBS and 1% P/S. Cell lines HEP 3B2.1-7, HEP G2, JHH2, JHH4, PLC/PRF/5 were grown in MEM supplemented with 1 mM sodium pyruvate, 2 mM L-glutamine, 1X NEAA solution, 10% FBS and 1% P/S and the SK-HEP-1 cell line was maintained in MEM supplemented with 1 mM sodium pyruvate, 2 mM L-glutamine, 1X NEAA solution, 20% FBS and 1% P/S. All cell lines were maintained at 37 °C and 5% CO_2_ in a humidified environment. Cell density was kept between 0.2 and 0.4 × 10^6^ cells per mL with 2–3 times per week medium renewal.

For proteomics and phosphoproteomics analysis, hepatic cell lines were seeded in petri dishes (between 0.3 and 3.74 × 10^6^ cells in 20 mL) and maintained in an incubator for 3–8 days at 37 °C and 5% CO_2_ in a humidified environment, until cell confluence reached 80% approximately. Medium was replaced with fresh complete medium 1.5 h prior to cell collection. Cells were subsequently washed three times with cold DPBS supplemented with 1 mM NaF and 1 mM Na_3_VO_4_ and lysed with 500 μL of urea buffer (8 M urea in 20 mM in HEPES, pH 8.0 supplemented with 1 mM NaF, 1 mM Na_3_VO_4_, 1 mM Na_4_P_2_O_7_ and 1 mM β-glycerophosphate). After cell collection with scrapers, lysates were snap frozen in protein LoBind tubes and stored at −80 °C for further sample preparation.

### Esophagus cancer cell lines

The Esophagus cancer cell lines KYSE-70, KYSE-140, KYSE-410, KYSE-450 and OE-19 were derived from male patients, while the cell lines COLO-680N, KYSE-150, KYSE-510, KYSE-520 and EO-33 were derived from female patients.

The KYSE-150 cell line was maintained in RPMI-1640 supplemented with 49% F12, 2% FBS and 1% P/S while KYSE-450 was grown in RPMI-1640 supplemented with 45% F12, 10% FBS and 1% P/S. All other esophagus cell lines were maintained in RPMI-1640 supplemented with 10% FBS and 1% P/S. All cell lines were maintained at 37 °C and 5% CO_2_ in a humidified environment. Cell density was kept between 0.1 and 0.25 × 10^6^ cells per mL.

For proteomics and phosphoproteomics experiments, esophagus cell lines were seeded in petri dishes (between 1.5 and 3.5 × 10^6^ cells in 10 mL) and maintained in an incubator overnight at 37 °C and 5% CO_2_ in a humidified environment. Then, cells were washed twice with cold DPBS supplemented with 1 mM NaF and 1 mM Na_3_VO_4_ and lysed in 500 μL of urea buffer (8 M urea in 20 mM in HEPES, pH 8.0 supplemented with 1 mM NaF, 1 mM Na_3_VO_4_, 1 mM Na_4_P_2_O_7_ and 1 mM β-glycerophosphate), snap frozen and stored at −80 °C until further processing.

### Sample preparation for phosphoproteomics and proteomics analysis

Phosphoproteomics and proteomics analysis was carried out as previously described^25,29,57^. AML cell pellets were lysed in 320 µL of urea buffer (8 M urea in 20 mM HEPES, pH: 8.0, supplemented with 1 mM Na_3_VO_4_, 1 mM NaF, 1 mM Na_4_P_2_O_7_ and 1 mM β-glycerophosphate). AML cell lysates were homogenised by sonication for 90 cycles (30 s on 30 s off) while thawed esophagus and hepatic cell lines were homogenised for 15 cycles (30 s on & 40 s off) in Diagenode Bioruptor^®^ Plus and insoluble material was removed by centrifugation.

Proteins were quantified using BCA protein assay. Then, 300 μg of protein were subjected to cysteine reduction and alkylation using sequential incubation with 10 mM dithiothreitol and 16.6 mM iodoacetamide for 1 h and 30 min, respectively, at 25 °C with agitation. Trypsin beads (50% slurry of TLCK-trypsin) were equilibrated with three washes with 20 mM HEPES (pH 8.0), the urea concentration in the protein suspensions was reduced to 2 M by the addition of 900 µL of 20 mM HEPES (pH 8.0), 100 μL of equilibrated trypsin beads were added and samples were incubated overnight at 37 °C. Trypsin beads were removed by centrifugation (2000 × *g* at 5 °C for 5 min) and samples were divided in 250 µg for phosphoproteomics analysis and 50 µg (200 µL) for proteomics analysis.

For phosphoproteomics analysis, peptide solutions were desalted using Oasis HLB cartridges (Waters) following the manufacturer’s indications. Briefly, cartridges were set in a vacuum manifold device and the pressure was adjusted to 5 mmHg. Then, cartridges were conditioned with 1 mL acetonitrile (ACN) and equilibrated with 1.5 mL of wash solution (0.1% trifluoroacetic acid (TFA), 2% ACN). Peptides were loaded in the cartridges and washed twice with 1 mL of wash solution. Finally, peptides were eluted with 500 µL of glycolic acid buffer 1 (1 M glycolic acid, 5% TFA, 50% ACN). Enrichment of phosphorylated peptides was performed with TiO_2_ Beads. Desalting eluents were normalized to 1 mL with glycolic acid buffer 2 (1 M glycolic acid, 5% TFA, 80% ACN) and incubated with 25 µl of TiO_2_ buffer (500 mg TiO_2_ beads in 500 µL of 1% TFA) for 5 min at room temperature. TiO_2_ beads were packed by centrifugation into empty spin columns previously washed with ACN. TiO_2_ beads were sequentially washed by centrifugation (1500 × *g* for 3 min) with 100 µL of glycolic acid buffer 2, ammonium acetate solution (100 mM ammonium acetate in 25% ACN) and three times with neutral solution (10% ACN). For phosphopeptide elution, spin tips were transferred to fresh tubes, 50 µL of elution solution 1 (5% NH_4_OH, 7.5% ACN) were added and tips were centrifuged at 1500 × *g* for 3 min. The elution step was repeated a total of four times. Finally, samples were snap frozen, dried in a SpeedVac vacuum concentrator and phosphopeptide pellets were stored at −80 °C.

For proteomics experiments, peptide solutions were desalted using C18 + carbon top tips (Glygen Corporation). The tips were conditioned twice with 200 µL of elution solution 2 (70/30 ACN/ H2O + 0.1% TFA) and equilibrated twice with 200 µL of wash solution. Sample were loaded into the top tips and washed twice with 200 µL of wash solution. For peptide elution, the tips were transferred to fresh tubes, and peptides were eluted three times with 250 µL of elution solution 2. In all desalting steps, tips were centrifuged at 1500 x *g* for 5 min at 5 °C. Eluted peptide solutions were dried in a SpeedVac vacuum concentrator and peptide pellets were stored at −80 °C.

### Mass spectrometry

To avoid batch effects, cells were grown and cell pellets (for AML) and protein extracts (for adherent cells) were collected and stored. Then, all samples were processed in consecutive days using the same buffer stocks and run in the same instrument in consecutive days in a random order. Note that the cell pellets and protein extracts for each of the three biological replicates per cell line were generated on different days to better capture the biological variability of the experiment. Samples were processed and run in the mass spectrometry in consecutive days in order to reduce technical variability.

Mass spectrometry for identification and quantification of proteins and phosphopeptides was carried out by LC-MS/MS as described before^29,57^. For phosphoproteomics analysis, peptide pellets were reconstituted in 13 µL of reconstitution buffer (20 fmol/µL enolase in 3% ACN, 0.1% TFA) and 5 µL were loaded onto an LC-MS/MS system. For proteomics analysis, peptide pellets were reconstituted in 10 µL of 0.1% TFA, 2 µL of this solution were further diluted in 18 µL of reconstitution buffer and 2 µL were injected into the LC-MS/MS system.

The LC-MS/MS platform consisted of a Dionex UltiMate 3000 RSLC coupled to Q Exactive^™^ Plus Orbitrap Mass Spectrometer (Thermo Fisher Scientific) through an EASY-Spray source. Mobile phases for the chromatographic separation of the peptides consisted of Solvent A (3% ACN; 0.1% FA) and Solvent B (99.9% ACN; 0.1% FA). Peptides were loaded in a μ-pre-column and separated in an analytical column using a gradient running from 3 to 23% B over 60 min (for phosphoproteomics) or 120 min (for proteomics). The UPLC system delivered a flow of 2 µL/min (loading) and 250 nL/min (gradient elution). The Q Exactive Plus operated a duty cycle of 2.1 s. Thus, it acquired full scan survey spectra (m/z 375–1500) with a 70,000 FWHM resolution followed by data-dependent acquisition in which the 15 most intense ions were selected for HCD (higher energy collisional dissociation) and MS/MS scanning (200–2000 m/z) with a resolution of 17,500 FWHM. A dynamic exclusion period of 30 s was enabled with m/z window of ±10 ppm. Mass spectrometry data collection was carried out using Thermo Scientific FreeStyle 1.4.

### Phosphopeptides and protein identification

Peptide identification from MS data was automated using Mascot Daemon 2.6.0 workflow in which Mascot Distiller v2.6.1.0 generated peak list files (MGFs) from RAW data and the Mascot search engine (v2.6) matched the MS/MS data stored in the MGF files to peptides using the SwissProt Database (SwissProt_2016Oct.fasta). Searches had a FDR of ~1% and allowed 2 trypsin missed cleavages, mass tolerance of ±10 ppm for the MS scans and ±25 mmu for the MS/MS scans, carbamidomethyl Cys as a fixed modification and PyroGlu on N-terminal Gln, oxidation of Met and phosphorylation on Ser, Thr, and Tyr as variable modifications (phosphorylation was only included for searches performed using phosphoproteomics data).

### Phosphopeptide and protein quantification

Identified peptides were quantified using a label-free procedure based on extracted ion chromatograms (XICs). Missing data points were minimized by constructing XICs across all LC-MS/MS runs for all the peptides identified in at least one of the LC-MS/MS runs^28^. XIC mass and retention time windows were ±7 ppm and ±2 min, respectively. Quantification of peptides was achieved by measuring the area under the peak of the XICs. Individual peptide intensity values in each sample were normalized to the sum of the intensity values of all the peptides quantified in that sample. Data points not quantified for a particular peptide were given a peptide intensity value equal to the minimum intensity value quantified in the sample divided by 10. For the phosphoproteomics experiments, we obtained a phosphorylation index (ppIndex) by summing the signals of all peptide ions containing the same modification site. For the proteomics experiment, protein intensity values were calculated by adding the intensities of all the peptides derived from a protein. Protein score values were expressed as the maximum Mascot protein score value obtained across samples.

### Data source and processing

Drug sensitivity and RNA-Seq data were sourced from PharmacoDB^32^. Proteomics and phosphoproteomics data was generated in-house for 26 AML, 10 esophagus and 12 HCC cell lines in house (see above) or obtained from^44,45,25^. Drug response, proteomics and phosphoproteomics datasets were normalized and proteomics and phosphoproteomics data were further normalized by centering and scaling. RNA-seq data was obtained as quantile normalized values.

### Empirical markers of sensitivity and resistance method

To reduce the dimensionality of the input datasets, we obtained EMDR using ensembles of statistical differences between cells resistant or sensitive to a given drug. For each drug, cell lines were separated into relative resistant or sensitive populations using the median drug response value (AAC) as cutoff. Resistant and sensitive populations were split into ten groups using the createMultiFolds caret function. AAC values in each of the sensitive groups were compared to each the resistant groups, leading to 100 comparisons. Resistant and sensitive samples in the repeats with *p* values < 0.05 between AAC values were used to generate markers. Linear models were generated and contrasts were computed for phosphorylation sites, proteins or transcripts across the sampled cell lines using the Limma package. The significance of these contrasts was assessed by empirical bayes statistics and *p* values adjusted for multiple testing using the Benjamini-Hochberg method. EMDRs were defined as significant if a fold value ±0.8 and *p* value < 0.05 were achieved in at least 80% of the repeats. Those found to be increased in sensitive cells relative to resistant were regarded as markers of sensitivity while those increased in resistant cells were considered to be resistant markers.

### DRUML method

Drug enrichment values were computed using custom in house scripts from the DRUMLR package^58^. For each drug and for each cell line, distance (*D*) values were computed by subtracting the median and 3rd quartiles of sensitivity markers expressions from resistance markers expressions. *D* values for each drug were correlated with all drug response data by spearman ranking and top correlated D values (ranging from 14 to 60, half of which had a positive, and the other half negative, correlation) were used as the input for ML models. The precise number of *D* values for model generation was determined as follows. We selected all the *D* values with spearman *p* value  < 0.05. If the number of correlated values was greater than 30 in a given direction (positive or negative correlation), then we selected the top 30 corrected values in such direction up to a maximum of 60. If the number of correlated D values was <7 in a given direction, then we selected the top 7 correlated *D* values in that direction. Models were built using the caret and h2o packages (see code availability section). Data was separated into training and validation populations at the cell line level with a partition ratio of 0.8 using the createDataPartion function in caret. *D* values were then normalized 0–1 before being used to build regression models using DL, nnet, Bayesian estimation in generalized linear models (bglm), random forest (rf), pls, principal components regression (pcr), svm and cubist ML models. The h2o R package was used for DL model generation and the caret R package for all other models. Each model underwent hyperparameter tuning with tenfold cross validation (repeated CV, *n* = 10, repeats = 3) using RMSE as the loss function except for pls and pcr models which were tuned using leave-one-out cross validation (code is provided in the GitHub repository for this project), and were subsequently validated using the validation data and verified using MS data from other laboratories. The code used for making and assessing the different learning models is provided in the DRUMLR package.

### Bioinformatics and statistics

Statistical analysis was carried out in R (v3.6) using base functions unless otherwise stated. ML model performance was evaluated using caret and R base functions. Ontology, pathway and kinase substrate enrichment analysis of EMDRs was evaluated using in-house R scripts using ontologies and pathways obtained from the literature^36,59–62^. Enrichment was calculated as [a/b]/[c/d], were a is number of EMDR that belong to a given set (pathway/ontology/downstream kinase substrate), b is number of EMDRs, c is number of proteins, phosphosites or transcripts that belong to such set in the background data and d is the size of the background data. The *p* values were obtained by the hypergeometric test and adjusted using the FDR method. Similarity scores between drugs were determined by correlating pathway enrichment values between pairs of drugs using the Pearson method in the ‘cor’ base R function. Pearson *R* values were scaled so that median and standard deviation of the distributing was 0 and 1 respectively, and *p* values determined by comparing each similarity score value against the distribution of all values using one sample *t*-test (R base function t.test). The R code used for these analyses is provided in the GitHub repository for this project.

### Reporting summary

Further information on research design is available in the Nature Research Reporting Summary linked to this article.

## Supplementary information

Reporting Summary

Supplementary Information

Supplementary Data 1

Supplementary Data 2

Supplementary Data 3

Supplementary Data 4

Supplementary Data 5

Supplementary Data 6

Supplementary Data 7

Supplementary Data 8

Supplementary Data 9

Description of Additional Supplementary Files

## Data Availability

The raw mass spectrometry proteomics and phosphoproteomics data generated during this study have been deposited to the ProteomeXchange Consortium via the PRIDE partner repository with the dataset identifier PXD019591 Project 10.6019/PXD019591). The processed omics datasets and EMDR data files are provided in https://github.com/CutillasLab/DRUML-publication-datasets. Drug sensitivity and RNA-seq data was sourced from PharmacoDB [https://zenodo.org/record/1038045#.YCw3K2j7SHs]^32^. Colorectal phosphoproteomics validation data were obtained from PRIDE dataset identifier PXD001550^44^. Proteomics validation data were drawn from PRIDE dataset identifier PXD013455^45^. AML patient phosphoproteomics validation data was obtained from PRIDE project PXD005978^25^. Drug information was sourced from DrugBank [https://go.drugbank.com/]^63^ and chEMBL [https://www.ebi.ac.uk/chembl/]^64^.

## Code availability

The following opensource R (v3.6) packages were used for DRUML model building and ontology/pathway and drug similarity analysis; foreach (v1.5.1), doParallel (v1.0.16), limma (v3.42.2), caret (v6.0-86), h2o (v3.32.0.1), Cubist (v0.2.3), pls (v2.7-3), glmnet (v4.0-2), kernlab (v0.9-29), ggdendro (v0.1.22) and randomForest (v4.6-14). The DRUMLR R package is accessible from https://github.com/CutillasLab/DRUMLR^58^ (10.5281/zenodo.4555600). The code for ontology/pathway and drug similarity analyses are available at https://github.com/CutillasLab/Term-Enrichment-Analysis (10.5281/zenodo.4562584).
