# Supplementary material for: Drug ranking using machine learning systematically predicts the efficacy of anti-cancer drugs
Source: Nat Commun. 2021 Mar 25;12:1850. doi: 10.1038/s41467-021-22170-8 (PMC7994645; doi:10.1038/s41467-021-22170-8)
Supplement: Supplementary file 1 — Reporting Summary [file 41467_2021_22170_MOESM1_ESM.pdf]

nature research

Corresponding author(s): Pedro R. Cutillas

Last updated by author(s): Feb 22, 2021

Reporting Summary

Nature Research wishes to improve the reproducibility of the work that we publish. This form provides structure for consistency and transparency in reporting. For further information on Nature Research policies, see our Editorial Policies and the Editorial Policy Checklist.

Statistics

For all statistical analyses, confirm that the following items are present in the figure legend, table legend, main text, or Methods section.

n/a	Confirmed	
☐	☒	The exact sample size (*n*) for each experimental group/condition, given as a discrete number and unit of measurement
☒	☐	A statement on whether measurements were taken from distinct samples or whether the same sample was measured repeatedly
☐	☒	The statistical test(s) used AND whether they are one- or two-sided<br>*Only common tests should be described solely by name; describe more complex techniques in the Methods section.*
☒	☐	A description of all covariates tested
☐	☒	A description of any assumptions or corrections, such as tests of normality and adjustment for multiple comparisons
☐	☒	A full description of the statistical parameters including central tendency (e.g. means) or other basic estimates (e.g. regression coefficient) AND variation (e.g. standard deviation) or associated estimates of uncertainty (e.g. confidence intervals)
☐	☒	For null hypothesis testing, the test statistic (e.g. *F*, *t*, *r*) with confidence intervals, effect sizes, degrees of freedom and *P* value noted<br>*Give P values as exact values whenever suitable.*
☒	☐	For Bayesian analysis, information on the choice of priors and Markov chain Monte Carlo settings
☒	☐	For hierarchical and complex designs, identification of the appropriate level for tests and full reporting of outcomes
☐	☒	Estimates of effect sizes (e.g. Cohen's *d*, Pearson's *r*), indicating how they were calculated

Our web collection on statistics for biologists contains articles on many of the points above.

Software and code

Policy information about availability of computer code

Data collection	ThermoScentific FreeStyle 1.4
Data analysis	Mascot Daemon 2.6.0, Mascot Distiller v2.6.1.0, Mascot search engine (v2.6), Pescal (beta.01), R-4.0.0, RStudio (Version 1.2.5042), DRUMLR (https://github.com/CutillasLab/DRUMLR.), Term Enrichment Analysis (https://github.com/CutillasLab/Term-Enrichment-Analysis), foreach (v1.5.1), doParallel (v1.0.16), limma (v3.44.2), caret (v6.0-86), h2o (v3.32.0.1), Cubist (v0.2.3), pls (v2.7-3), glmnet (v4.0-2), kernlab (v0.9-29), ggdendro (v0.1.22) and randomForest (v4.6-14).

For manuscripts utilizing custom algorithms or software that are central to the research but not yet described in published literature, software must be made available to editors and reviewers. We strongly encourage code deposition in a community repository (e.g. GitHub). See the Nature Research guidelines for submitting code & software for further information.

Data

Policy information about availability of data

All manuscripts must include a data availability statement. This statement should provide the following information, where applicable:

- Accession codes, unique identifiers, or web links for publicly available datasets
- A list of figures that have associated raw data
- A description of any restrictions on data availability

The raw mass spectrometry proteomics and phosphoproteomics data generated during this study have been deposited to the ProteomeXchange Consortium via the PRIDE partner repository with the dataset identifier PXD019591 (Project DOI: 10.6019/PXD019591).
The processed omics datasets and EMDR data files are provided in https://github.com/CutillasLab/DRUML-publication-datasets.
Drug sensitivity and RNA-seq data was sourced from PharmacoDB: https://zenodo.org/record/1038045#.YCw3K2j7SHs
Colorectal phosphoproteomics validation data were obtained from PRIDE dataset identifier PXD001550 :https://www.ebi.ac.uk/pride/archive/projects/PXD001550

Proteomics validation data were drawn from PRIDE dataset identifier PXD013455: https://www.ebi.ac.uk/pride/archive/projects/PXD013455.
AML patient phosphoproteomics validation data was obtained from PRIDE project PXD005978: https://www.ebi.ac.uk/pride/archive/projects/PXD005978.
Drug information was sourced from DrugBank and chEMBL: https://go.drugbank.com/ and https://www.ebi.ac.uk/chembl/

Field-specific reporting

Please select the one below that is the best fit for your research. If you are not sure, read the appropriate sections before making your selection.

[x] Life sciences [ ] Behavioural & social sciences [ ] Ecological, evolutionary & environmental sciences

For a reference copy of the document with all sections, see nature.com/documents/nr-reporting-summary-flat.pdf

Life sciences study design

All studies must disclose on these points even when the disclosure is negative.

Sample size	No sample size calculation was performed. Sample sizes were determined by cell line availability at the start of the project and these deemed sufficient as they produced learning models with reasonable low error.
Data exclusions	No data were excluded from the analysis.
Replication	Samples were analyzed in triplicate (independent cell cultures per cell line). Reproducibility was assessed by hierarchical clustering showing that replicates grouped together.
Randomization	Samples were grouped into training and testing test using the createPartitition function in the caret package. This function balances the class distributions within the splits. Partitions were specific for each drug.
Blinding	Investigators were not blinded to the study design because model generation was carried out using supervised methods.

Reporting for specific materials, systems and methods

We require information from authors about some types of materials, experimental systems and methods used in many studies. Here, indicate whether each material, system or method listed is relevant to your study. If you are not sure if a list item applies to your research, read the appropriate section before selecting a response.

Materials & experimental systems

n/a	Involved in the study	
[x]	[ ]	Antibodies
[ ]	[x]	Eukaryotic cell lines
[x]	[ ]	Palaeontology and archaeology
[x]	[ ]	Animals and other organisms
[x]	[ ]	Human research participants
[x]	[ ]	Clinical data
[x]	[ ]	Dual use research of concern

Methods

n/a	Involved in the study	
[x]	[ ]	ChIP-seq
[x]	[ ]	Flow cytometry
[x]	[ ]	MRI-based neuroimaging

Eukaryotic cell lines

Policy information about cell lines

Cell line source(s)	DSMZ-German Collection of Microorganisms and Cell Cultures GmbH and American Type Culture Collection (ATCC)<br><br>The AML cell lines AML-193, CMK, K-052, Kasumi-1, KG-1, HEL, ME-1, ML-2, MOLM-13, MONO-MAC-6, MV4-11, OCI-AML2, OCI-AML3, OCI-AML5, P31/FUJ, PL-21, SIG-M5, SKM-1 and THP-1 were from GmbH.<br><br>The hepatic cancer cell lines (HEP 3B2.1-7, HEP G2, JHH2, JHH4, SK-HEP-1, SNU182, SNU-398, SNU-423, SNU-449 and SNU-475) and the Esophagus cancer cell lines (KYSE-70, KYSE-140, KYSE-410, KYSE-450, OE-19, COLO-680N, KYSE-150, KYSE-510, KYSE-520 and EO-33) were from ATCC.
Authentication	Cell lines were obtained from repositories with certificate of analysis, and used without further authentication.
Mycoplasma contamination	Cells tested negative for mycoplasma contamination.
Commonly misidentified lines (See ICLAC register)	No commonly misidentified cell lines were used in the study.

April 2020
