# Supplementary material for: Drug ranking using machine learning systematically predicts the efficacy of anti-cancer drugs
Source: Nat Commun. 2021 Mar 25;12:1850. doi: 10.1038/s41467-021-22170-8 (PMC7994645; doi:10.1038/s41467-021-22170-8)
Supplement: Supplementary file 2 — Supplementary Information [file 41467_2021_22170_MOESM2_ESM.docx]

**Supplementary Information**

**Drug ranking using machine learning systematically predicts the efficacy of anti-cancer drugs**

Henry Gerdes, Pedro Casado, Arran Dokal, Maruan Hijaz, Nosheen Akhtar, Ruth Osuntola, Vinothini Rajeeve, Jude Fitzgibbon, Jon Travers, David Britton, Shirin Khorsandi & Pedro R. Cutillas

**Table of Supplementary Data files**

| **Data #** | **Description** |
| --- | --- |
| Supplementary Data 1 | Details of cell lines and study design. |
| Supplementary Data 2 | Processed phosphoproteomics data |
| Supplementary Data 3 | Processed proteomics data |
| Supplementary Data 4 | Empirical markers of drug response |
| Supplementary Data 5 | Results of systematic ontology and pathway analysis of empirical drug response markers |
| Supplementary Data 6 | Drug similarity scores. Drug similarity indices calculated by comparing enriched ontologies in empirical drug response markers for all drugs |
| Supplementary Data 7 | Accuracy of ML models in validation dataset. machine model. Performance was determined by spearman rank correlation and root mean square error analysis. |
| Supplementary Data 8 | Accuracy of Drug Ranking Prediction in verification CRC cells. Machine learning model performance was determined in independent data from colorectal cancer cell lines as shown in Figure 5 of main paper. |
| Supplementary Data 9 | Accuracy of Drug Ranking Prediction in verification 48 Cancer Cell Lines. Machine learning model performance was determined in independent data from 48 cancer cell lines as shown in Figure 6 of main paper. |
| Data are also provided in the following GitHub repository:  [https://github.com/CutillasLab/DRUML-publication-datasets](about:blank) | |


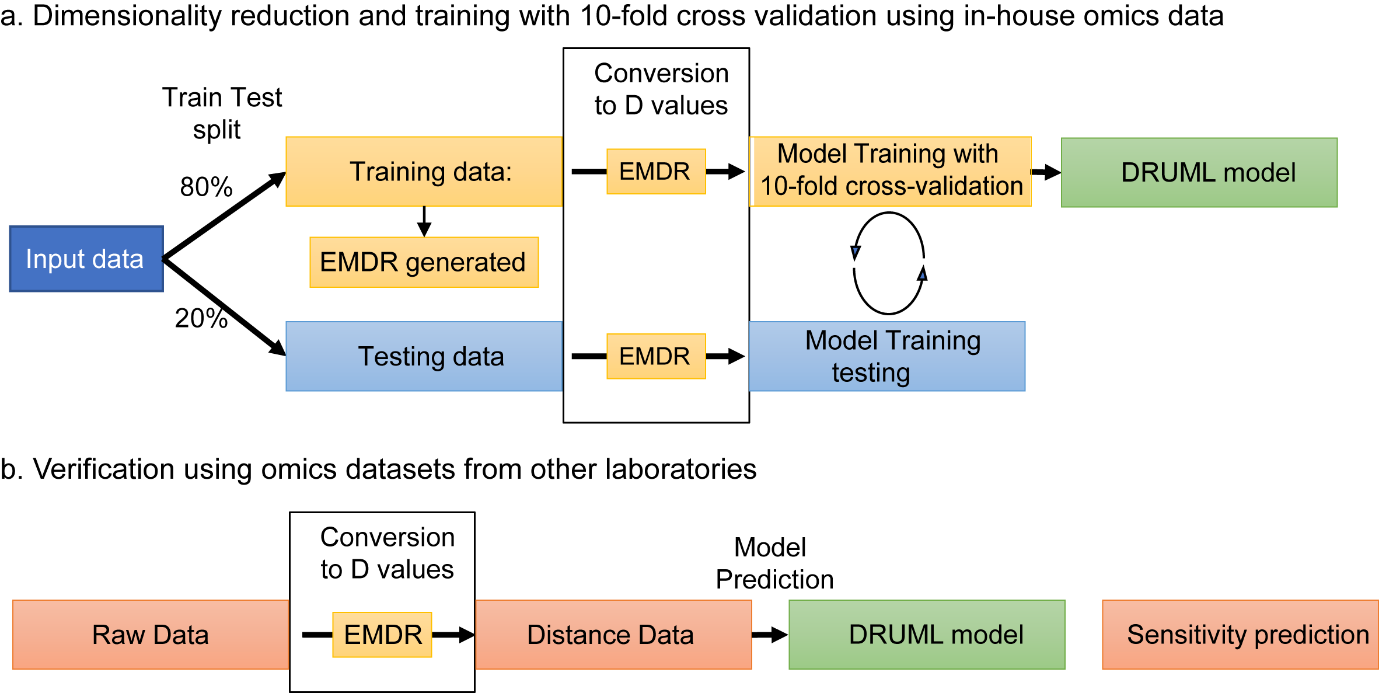


**Supplementary Figure 1. Scheme for model training, validation and verification**

**a** Proteomics, phosphoproteomic or transcriptomic data (n=48) were split into training (yellow) and test (blue) sets by sample to make drug ranking using machine learning (DRUML) models (green). The training data was used to identify markers of drug responses (EMDRs) for 466 drugs, thereby reducing the number of features used to build models. These signatures were then averaged into a distance metric (D) and these were used for machine learning model training. **b** The models were verified using phosphoproteomics and proteomics datasets obtained from other laboratories (orange). This process consisted of converting the omics data into D values using the EMDRs identified in the training process (a), which were used as input features for drug response prediction.


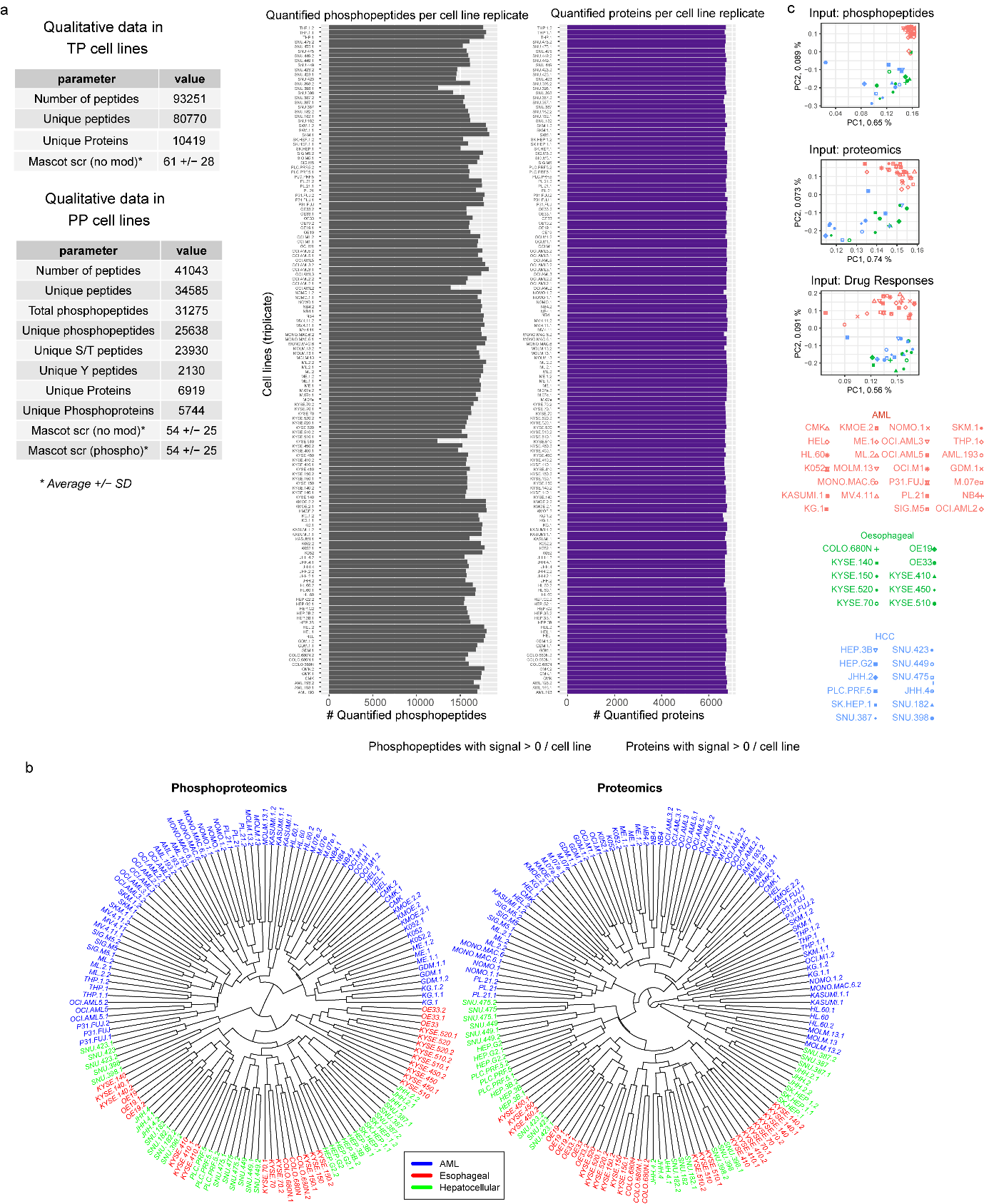


**Supplementary Figure 2. Qualitative assessment of proteomics and phosphoproteomics data**

**a** Number of peptides, phosphopeptides and proteins identified from the proteomic analysis of 48 acute myeloid leukemia, esophageal and hepatocellular carcinoma cell lines shown in Figure 1. Number of phosphopeptides (left) and unmodified proteins (right) quantified in each of the replicates from all the 48 analyzed cell lines. **b** Unsupervised hierarchical clustering of cell lines based on phosphoproteomics and proteomics data. **c** Principal components analysis of proteomics, phosphoproteomics and drug response datasets of the named cell lines used to train DRUML.

**
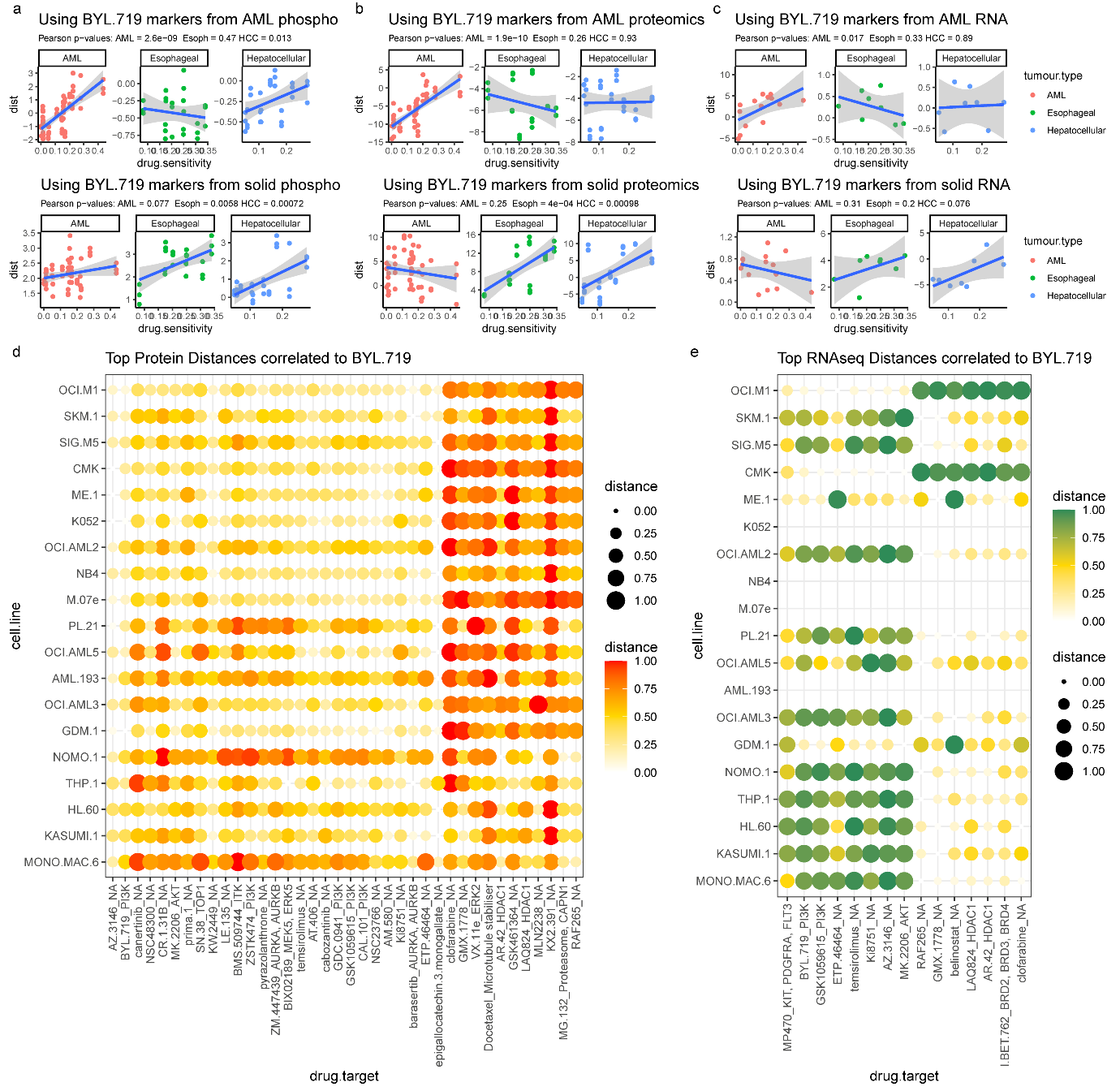
Supplementary Figure 3. Dimensionality reduction using empirical markers of drug responses**

**a, b, c** Associations between drug sensitivity of cell lines to BYL-719 and the distance metric D obtained from phosphoproteomics (a), proteomics (b) of RNA-seq (c) data. Data were split into AML (n=19), esophageal (n=7) and hepatocellular(n=10) cancer subsets. *D* values were computed by combining the expression of empirical markers of sensitivity and resistance as shown in Fig 1a and in the main text. p-values were generated by Pearson test. Linear regression lines are displayed with standard error margins shown as grey plot regions. **d, e** Mean expression top drug marker distances values which correlate both positively and negatively with drug sensitivity to BYL-719 obtained from proteomics (d) or RNA-seq (e) data. Rows are organized in order of BYL-719 sensitivity (AAC). Dot color intensities and sizes are proportional to distance values normalized 0 to 1.


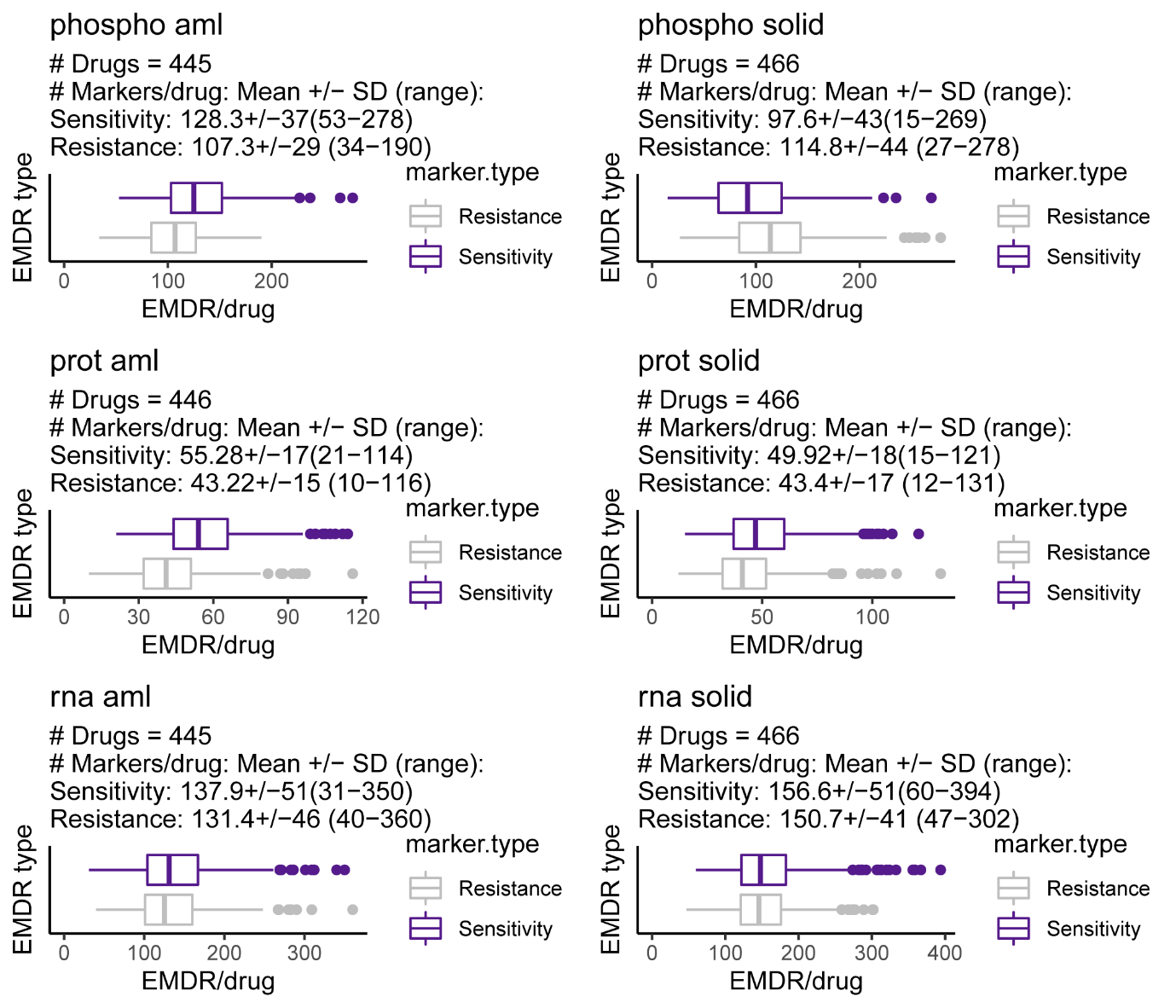


**Supplementary Figure 4. Number of empirical markers of drug responses (EMDRs) identified per drug**

Markers of resistance and sensitivity refer to phosphorylation sites, proteins or transcripts increased (grey) or decreased (purple) in cells resistant to a given drug, respectively. Data were split into AML (n=26) and solid (n=22) cancer subsets. Boxplots show median centers, interquartile box boundaries and range upper and lower hinges.

**
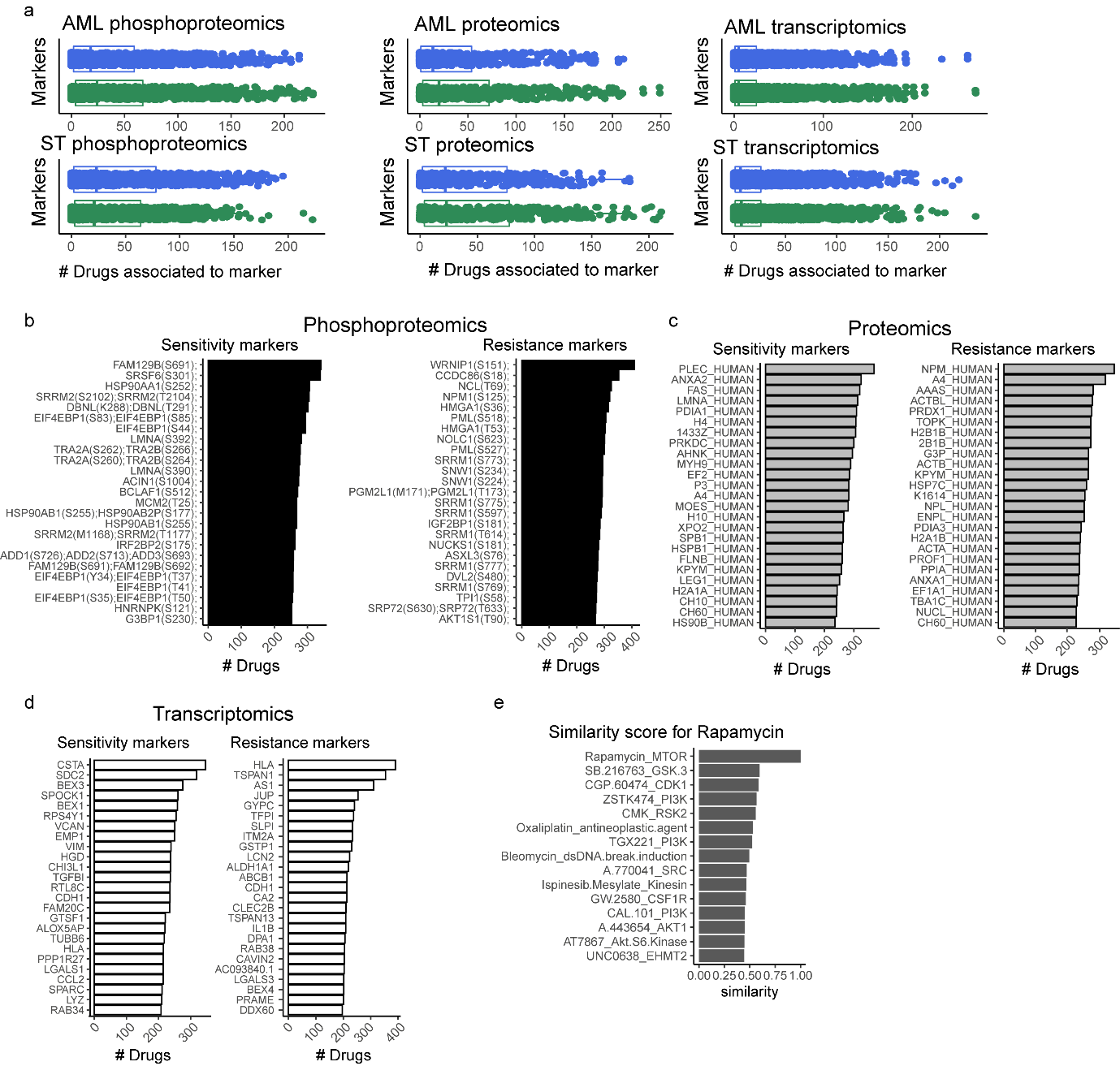
**

**Supplementary Figure 5. Overview of systematic empirical markers of responses to >400 drugs**

**a** Distribution of number of drugs for which phosphorylation sites (left), proteins (middle), and transcripts (right) were identified as empirical drug response markers. These markers were generated using AML (n=26) and solid (n=22) cancer omics data. Sensitive markers are shown in blue while resistant markers are shown in green. Boxplots show median centers, interquartile box boundaries and range upper and lower hinges. **b, c, d** Bar charts of total frequency of feature presence in empirical markers of drug response. The top 25 most frequently identified as phosphorylation site, protein and transcript markers of sensitivity or resistance are shown. **e** The top 20 most similar drugs associated Rapamycin, as measured by similarity score.


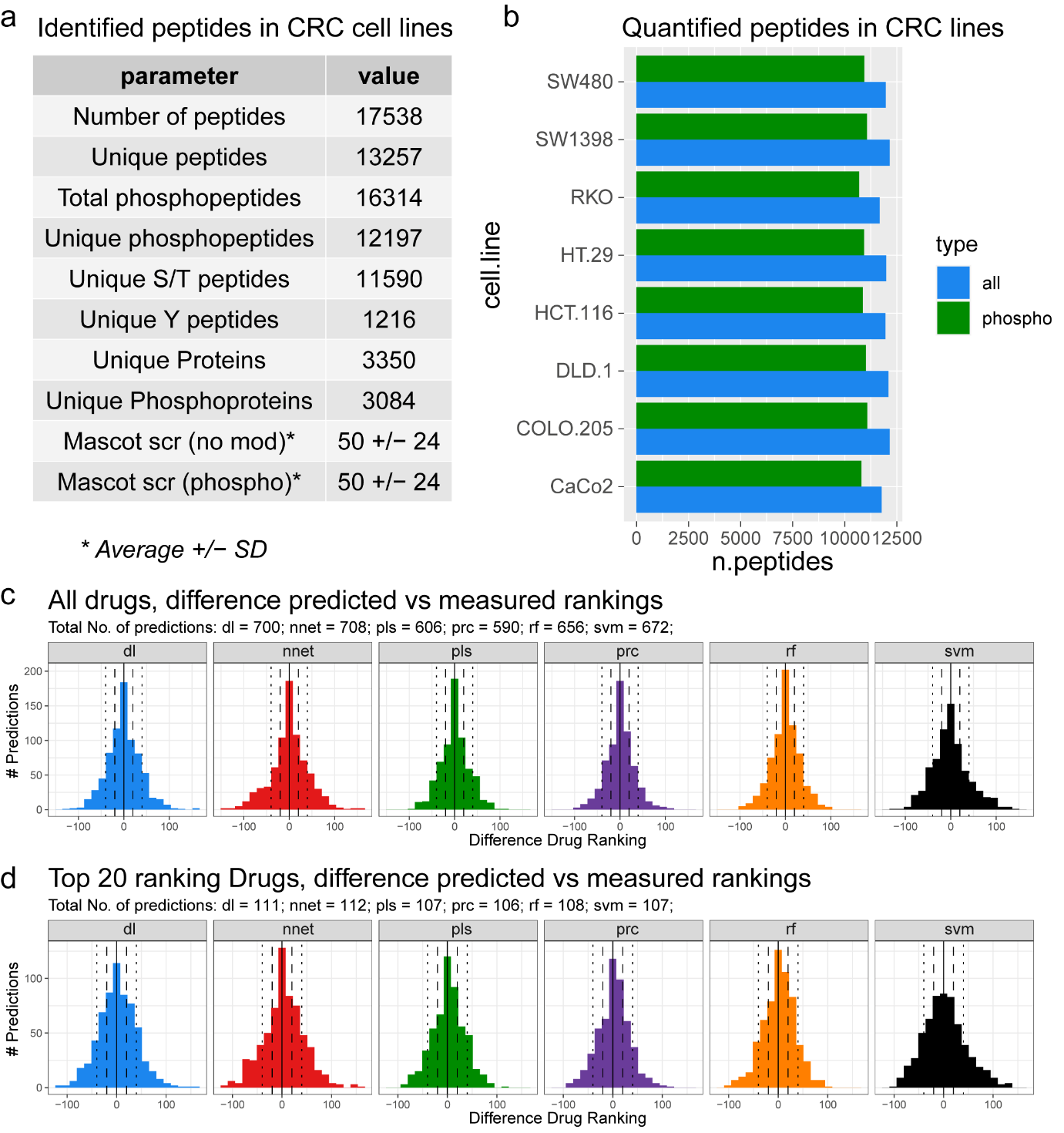


**Supplementary Figure 6. DRUML prediction of drug response rakings from an independent phosphoproteomics dataset**

**a, b** Qualitative assessment of phosphoproteomics data from 8 colorectal cancer cell lines obtained from Piersma *et al*. **c, d** Differences between predicted and measured drug ranking positions from all 389 drugs for which predictions were obtained (c) or the top 20 most sensitive drugs for each cell line (d). Drug ranking predictions from all samples were pooled. Dashed and dotted lines indicate 20 and 50 position difference error in drug ranking, respectively. Learning algorithms were random forest (rf), cubist, bayesian estimation of generalized linear models (bglm), partial least squares (pls), principal component regression (pcr), support vector machine (svm), deep learning (dl) and neural network (nnet).


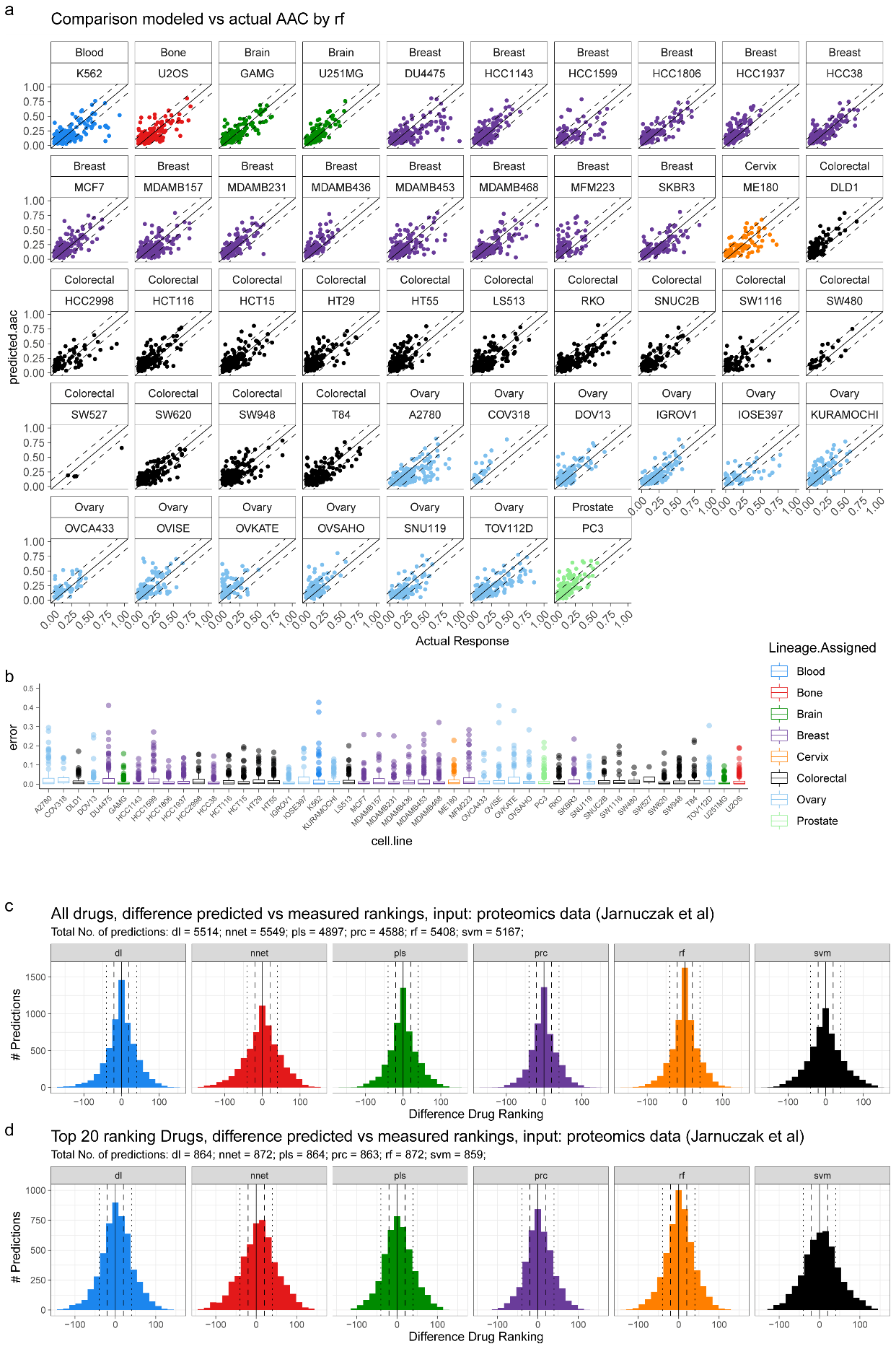


**Supplementary Figure 7. Accuracy assessment of DRUML to rank drugs based on efficacy using independent proteomics datasets derived from 47 tumor models and 8 pathologies**

**a** Results of DRUML-based drug response predictions using random forest (rf) learning models in proteomics verification data (n=47 cell lines). Solid line displays 0% absolute error threshold while dotted lines represent 10% absolute error margins. **b** Squared error of drug predictions across cell lines. Boxplots show median centers, interquartile range boxes and range hinges. **c**, **d** Differences between predicted and measured drug ranking positions from all 389 drugs for which predictions could be derived by at least one learning models (c) or the top 20 most sensitive drugs for each cell line (d). Drug ranking predictions from all samples were pooled. Dashed and dotted lines indicate 20 and 40 position difference error in drug ranking, respectively. Learning algorithms were random forest (rf), cubist, bayesian estimation of generalized linear models (bglm), partial least squares (pls), principal component regression (pcr), support vector machine (svm), deep learning (dl) and neural network (nnet).
