# Supplementary material for: Drug ranking using machine learning systematically predicts the efficacy of anti-cancer drugs
Source: Nat Commun. 2021 Mar 25;12:1850. doi: 10.1038/s41467-021-22170-8 (PMC7994645; doi:10.1038/s41467-021-22170-8)
Supplement: Supplementary file 12 — Description of Additional Supplementary Files [file 41467_2021_22170_MOESM12_ESM.pdf]

Title: Supplementary Data 1
Description: Details of cell lines and study design.

Title: Supplementary Data 2
Description: Processed phosphoproteomics data

Title: Supplementary Data 3
Description: Processed proteomics data

Title: Supplementary Data 4
Description: Empirical markers of drug response

Title: Supplementary Data 5
Description: Results of systematic ontology and pathway analysis of empirical drug response markers

Title: Supplementary Data 6
Description: Drug similarity scores. Drug similarity indices calculated by comparing enriched ontologies in empirical drug response markers for all drugs

Title: Supplementary Data 7
Description: Accuracy of ML models in validation dataset. machine model. Performance was determined by spearman rank correlation and root mean square error analysis.

Title: Supplementary Data 8
Description: Accuracy of Drug Ranking Prediction in verification CRC cells. Machine learning model performance was determined in independent data from colorectal cancer cell lines as shown in Figure 5 of main paper.

Title: Supplementary Data 9
Description: Accuracy of Drug Ranking Prediction in verification 48 Cancer Cell Lines. Machine learning model performance was determined in independent data from 48 cancer cell lines as shown in Figure 6 of main paper.
